# Single-cell RNA-seq reveals immune cell heterogeneity and increased Th17 cells in human fibrotic skin diseases

**DOI:** 10.3389/fimmu.2024.1522076

**Published:** 2025-01-13

**Authors:** Cheng-Cheng Deng, Xue-Yan Xu, Yan Zhang, Long-Can Liu, Xuan Wang, Jun-Yi Chen, Liu-Yi Yao, Ding-Heng Zhu, Bin Yang

**Affiliations:** Dermatology Hospital, Southern Medical University, Guangzhou, China

**Keywords:** immune cell, Th17 cell, fibrotic skin diseases, keloid, macrophage, dendritic cell, IL-17

## Abstract

**Background:**

Fibrotic skin disease represents a major global healthcare burden, characterized by fibroblast hyperproliferation and excessive accumulation of extracellular matrix components. The immune cells are postulated to exert a pivotal role in the development of fibrotic skin disease. Single-cell RNA sequencing has been used to explore the composition and functionality of immune cells present in fibrotic skin diseases. However, these studies detected the gene expression of all cells in fibrotic skin diseases and did not enrich immune cells. Thus, the precise immune cell atlas in fibrotic skin diseases remains unknown. In this study, we plan to investigate the intricate cellular landscape of immune cells in keloid, a paradigm of fibrotic skin diseases.

**Methods:**

CD45^+^ immune cells were enriched by fluorescence-activated cell sorting. Single-cell RNA sequencing was used to analyze the cellular landscape of immune cells in keloid and normal scar tissues. Ki-67 staining, a scratch experiment, real-time PCR, and Western blotting were used to explore the effect of the Th17 cell supernatant on keloid fibroblasts.

**Results:**

Our findings revealed the intricate cellular landscape of immune cells in fibrotic skin diseases. We found that the percentage of Th17 cells was significantly increased in keloids compared to normal scars. All the subclusters of macrophages and dendritic cells (DCs) showed similar proportions between keloid samples and normal scar samples. However, upregulated genes in keloid M1 macrophages, M2 macrophages, and cDC2 are associated with the MHC class II protein complex assembly and antigen assembly, indicating that macrophages and cDC2 are active in keloids. Functional studies suggested that the supernatant of Th17 cells could promote proliferation, collagen expression, and migration of keloid fibroblasts through interleukin 17A. Importantly, increased Th17 cells are also found in other fibrotic skin diseases, such as hypertrophic scars and scleroderma, suggesting this represents a broad mechanism for skin fibrosis.

**Conclusion:**

In summary, we built a single-cell atlas of fibrotic skin diseases in this study. In addition, we explored the function of Th17 cell-fibroblast interaction in skin fibrosis. These findings will help to understand fibrotic skin disease pathogenesis in depth and identify potential targets for fibrotic skin disease treatment.

## 1 Introduction

Fibrosis is a condition that is characterized by fibroblast proliferation and excessive accumulation of extracellular matrix components (1, 2). Fibrosis contributes to a high level of morbidity and mortality worldwide and can lead to progressive tissue scarring and organ dysfunction (1–3). Fibrotic skin diseases are characterized by the accumulation of extracellular matrix components in the dermis and include hypertrophic scars, keloids, scleroderma, and graft-vs.-host diseases (4–8). Studies have indicated a correlation between the development of fibrotic skin diseases and genetic predisposition, tissue tension, aberrant collagen synthesis and degradation processes, inflammatory responses, and immune dysregulation (3, 6, 7). However, the precise underlying pathogenesis of fibrotic skin diseases remains elusive, and radical treatments are still lacking.

The immune response is postulated to exert a pivotal role in the occurrence and progression of fibrotic skin diseases (6–10). A substantial infiltration of immune cells is observed within fibrotic skin diseases, and these cells potentially influence the development of fibrotic skin disease lesions through the release of inflammatory mediators and the modulation of extracellular matrix synthesis. Furthermore, it has been demonstrated that immune cells occupy a pivotal position in regulating the aberrant behavior exhibited by fibroblasts in fibrotic skin diseases (6, 7, 9, 11, 12). Single-cell RNA sequencing (scRNA-seq) has been used to explore the composition and functionality of immune cells present in fibrotic skin diseases, such as in keloids and scleroderma (13–15). However, these studies detected the gene expression of all cells in fibrotic skin diseases and did not enrich immune cells. Most of the cells in these single-cell RNA sequencing studies were keratinocytes, fibroblasts, and vascular endothelial cells, and the proportion of immune cells was low in the results (13–15). We need to enrich immune cells in single-cell RNA sequencing studies of fibrotic skin diseases to get a more precise immune cell atlas for fibrotic skin diseases.

Th17 cells are a lineage of CD4^+^ T helper cells. Th17 cells have been implicated in numerous inflammatory diseases, including Crohn’s disease, psoriasis, multiple sclerosis, rheumatoid arthritis, and inflammatory bowel disease (16–18). The pro-inflammatory cytokines derived from Th17 cells, including interleukin 17A (IL-17A), IL-17F, IL-21, IL-22, and IL-26, play crucial roles in the pathogenesis of these diseases (16–18). It has been observed that the inhibition of Th17 cell differentiation leads to a downregulation of IL-17A expression, subsequently mitigating hepatic fibrosis and pulmonary fibrosis (19, 20). It has been discovered that IL-17A secreted by Th17 cells augments the release of pro-inflammatory chemokines, including monocyte chemoattractant protein (MCP)-1 and IL-8, from dermal fibroblasts in systemic sclerosis. This, in turn, exerts a profound impact on the remodeling of the extracellular matrix (21, 22). These comprehensive investigations have established a link between Th17 cells and fibrotic diseases. However, the specific role and function of Th17 cells in fibrotic skin diseases are not fully understood.

In this study, we isolated CD45^+^ cells from keloids, a paradigm of fibrotic skin diseases, using fluorescence-activated cell sorting (FACS) and performed single-cell RNA sequencing analysis. Our results revealed the intricate cellular landscape of immune cells in keloids. Compared to normal scar tissue, the percentage of Th17 cells was significantly increased in keloids. Further functional studies revealed that Th17 cells promote the proliferation, collagen expression, and migration of keloid fibroblasts by secreting IL-17A. Importantly, increased Th17 cells were also found in other fibrotic skin diseases, such as hypertrophic scars and scleroderma, suggesting this represents a broad mechanism for skin fibrosis. These findings will help us more thoroughly understand the pathogenesis of fibrotic skin diseases and provide potential targets for therapies for fibrotic skin diseases.

## 2 Materials and methods

### 2.1 Sample preparation and tissue dissociation

This study was approved by the Medical and Ethics Committees of Dermatology Hospital, Southern Medical University, and each patient signed an informed consent form before participating in this study. Keloid tissues were harvested during plastic surgery from three patients confirmed to have clinical evidence of keloid (**Supplementary Table S1**). Normal scar tissue was obtained from three patients who underwent elective scar resection surgery (**Supplementary Table S1**). The tissues were washed with PBS on ice and the fat tissue was removed. The tissue samples were then cut into 1 cm^2^ pieces in a digestion medium composed of 2.5 mg/ml Dispase II (Roche, USA, 04942078001) in PBS and incubated at 37°C for 2h. After removing the epidermis, the dermal portion was further cut and digested in 2.5 mg/ml collagenase IV (Yeasen Biotechnology, China; 40510ES60) at 37°C for 2h. The cell suspension was filtered through a 70μm-cell strainer, and then the enzymes were neutralized with buffer (PBS with 1% fetal bovine serum). The cells were centrifuged at 2,000 rpm for 10 min at 4°C and resuspended in buffer (PBS with 1% FBS). We then sorted the CD45^+^ immune cells and constructed scRNA-seq libraries.

### 2.2 Single-cell cDNA and library preparation

Single-cell cDNA, library preparation, and 3′-end single-cell RNA sequencing were performed by Novogene (Beijing, China). For experiments using the 10×Genomics platform, the Chromium Single Cell 3′ Library and Gel Bead Kit v3.1, Chromium Single Cell 3′ Chip Kit v3.1, and Chromium i7 Multiplex Kit were used according to the manufacturer’s instructions in the Chromium Single Cell 3′ Reagents Kits v3.1 User Guide. The single-cell suspension was washed twice with 1×PBS + 0.04% BSA. The cell number and concentration were confirmed using a TC20™ Automated Cell Counter.

Approximately 10,000 cells were immediately subjected to the 10×Genomics Chromium Controller machine for Gel Beads-in-Emulsion (GEM) generation. mRNA was prepared using 10×Genomics Chromium Single Cell 3′ reagent kits (V3 chemistry). During this step, cells were partitioned into the GEMs along with gel beads coated with oligos. These oligos provide poly-dT sequences to capture mRNAs released after cell lysis inside the droplets and cell-specific and transcript-specific barcodes (16 bp 10×barcodes and 10 bp unique molecular identifiers (UMIs), respectively).

After RT-PCR, cDNA was recovered, purified, and amplified to generate sufficient quantities for library preparation. Library quality and concentration were assessed using an Agilent Bioanalyzer 2100. Libraries were run on the Novaseq 6000 for Illumina PE150 sequencing.

### 2.3 Single-cell RNA-sequence data processing

The 10×Genomics Cell Ranger toolkit (v6.1.2) was used to process 10×Genomics raw data for read alignment and UMI matrix generation. Reads were aligned to the human reference genome (GRCh38) downloaded from the 10×Genomics official website with the STAR algorithm. The aligned reads were quantified as a gene expression matrix based on the number of UMIs detected in individual cells. Filtered gene-cell UMI matrices were generated for further single-cell analysis.

Low-quality cells that expressed fewer than 200 genes or more than 4,000 genes and more than 10% mitochondrial gene expression were eliminated. The R package DoubletFinder (v2.0.3) was applied to filter doublets. After removing low-quality cells and doublets, R package Seurat (v4.0.3) was used for unsupervised clustering of individual cells. First, a global-scaling normalization method, LogNormalize, that normalizes the gene expression measurements for each cell by the total number of UMIs in single cells and multiplied by a scaling factor of 10000 was used. After log-transformation, the top 2,000 highly variable genes were detected and principal component analysis (PCA) was performed for downstream unsupervised clustering analysis. The Louvain algorithm was adopted to cluster individual cells based on the top 30 PCs and the identified clusters were visualized with Uniform Manifold Approximation and Projection (UMAP). The accuracy of single-cell analysis can be affected by batch effects, and the canonical correlation analysis (CCA) method was applied based on the top 30 PCs with the default parameters for batch correction.

### 2.4 Gene signature scores

To assist in the identification of subpopulations of CD4 and CD8 T cells, we downloaded the sets of gene signatures associated with CD4 and CD8 T cells from the literature (23) and calculated functional signature scores for each cell with the AddModuleScore function in the Seurat package to illustrate the functional properties of each cell type.

To assign M1/M2 polarization estimates to the macrophage cells, we applied the AddModuleScore function in the Seurat package. The gene sets associated with M1 and M2 polarization were obtained from Sun et al. (24).

### 2.5 Functional enrichment analysis

Differentially expressed genes (DEGs) were identified using the FindMarkers function, implemented in the Seurat package, with the Wilcoxon rank sum test with the following criteria: log-scaled fold change ≥ 0.25 and P value < 0.05. Gene Ontology (GO) and Kyoto Encyclopedia of Genes and Genomes (KEGG) pathway analyses were performed using the clusterProfiler (v4.11.0) package based on the upregulated genes and downregulated genes. Pathways with adjusted P < 0.05 were considered significant.

### 2.6 Cell-cell interactions analysis

To investigate the cell-cell interactions between different cell types in the normal scar samples and keloid samples, cellular spatial organization mapper (CSOmap) software (v1.0) was used to identify ligand-receptor pairs. CSOmap was used to construct a three-dimensional (3D) pseudo space and infer the cell-cell interactions based on scRNA-seq data. CSOmap combined the gene expression data of single cells with prior knowledge of signaling and gene regulatory networks. FANTOM5, a human ligand-receptor interaction database, was used to combine immune-associated chemokines, cytokines, costimulators, coinhibitors, and their receptors to estimate the cell-cell affinity matrix. The contribution of each L-R pair to the cell-cell affinity can provide clues to highlight important LR pairs underlying the cellular interactions.

### 2.7 Immunofluorescence staining

Human skin biopsies (**Supplementary Table S2**) were submerged in 4% paraformaldehyde for 24h at room temperature. The samples were dehydrated in gradient alcohol and embedded in paraffin according to standard protocols. Samples were sectioned at 4μm thickness and then incubated at 75℃ for 20min. The sections were deparaffinized with environmental dewaxling dip wax transparentize solution (Bioshap, China, 22181809) and rehydrated in 95% alcohol. The sections were placed with high-pH repair buffer (GeneTech, China; GT102410) in a 95℃ water bath for 20 min with a microwave. After overnight incubation at 4℃ with rabbit anti-IL-17A (Santa Cruz, sc-374218) and mouse anti-CD4 (Abcam, ab183685), sections were washed thrice with PBS and treated with 1:1000 diluted anti-rabbit Alexa Flour 488 (Abcam, ab150113) and anti-mouse Alexa Flour555 (Abcam, ab150110), and conjugated for 1 h at room temperature. After three washes with PBS, counterstaining of cell nuclei was performed using DAPI (Beyotime, China, P0131). Images were taken using a Nikon A1 confocal laser-scanning microscope.

### 2.8 Th17 cells polarizing

Peripheral blood mononuclear cells (PBMCs) were isolated from keloid patients’ whole blood by centrifugation in a density gradient medium (Ficoll-PaqueTM Plus, Cytiva, 17144003). The cells were resuspended at a concentration of 5×10^7^ cells/mL in buffer (PBS containing 2% fetal bovine serum and 1 mM EDTA). Naïve CD4^+^ T cells (purity >99%) were isolated using an EasySep™ Human Naïve CD4^+^ T Cell Isolation Kit II (Stemcell, 17555). Purified naïve T cells obtained as described above were cultured in a Th17-polarizing medium for 7 days to induce Th17 cells. The Th17-polarizing medium contained anti-CD3 Ab (2μg/mL, OKT-3; BioLegend), anti-CD28 Ab (1μg/mL, OKT-3, BioLegend), IL2 (10ng/ml,PeproTech), IL6 (20ng/mL, R&D Systems), TGF-β1 (10ng/mL, R&D Systems), IL1β (10ng/mL, PeproTech), IL23 (10ng/mL, PeproTech), anti-IL4 Ab (10μg/mL, BioLegend), and anti-IFN-γ Ab (10μg/mL, BioLegend).

### 2.9 Real-time quantitative PCR

RNA extraction from cells was performed using TRIzol Reagent (Invitrogen, Life Technologies, USA) according to the manufacturer’s instructions. 1μg of RNA fraction was reverse transcribed to cDNA using PrimeScript™ RT Master Mix (Takara, Dalian, China). qRT-PCR was conducted using a BIO-RAD CFX Connect Real-time PCR Detection System and primers and templates mixed with SYBR Premix Ex Taq II (Vazyme, Nanjing, China). Threshold cycle (CT) values were used to calculate the fold change using the 2^-ΔΔCT^ method. The relative mRNA expression was normalized to the *GAPDH* gene. Gene-specific primer pairs were designed with Primer Premier 5.0 software (**Supplementary Table S3**).

### 2.10 Western blot

The cells were washed once with ice-cold PBS and lysed with chilled RIPA buffer containing protease inhibitors. Cell lysates were separated by 10% SDS-PAGE (Bio-Rad) and then transferred from the gel to 0.45 um polyvinylidene difluoride membranes (Millipore, Billerica, USA). Page Ruler Plus Prestained Protein Ladder (Fermentas, Hanover, USA) was used to confirm protein electrophoresis and transferring. After blocking in a solution of 5% non-fat dry milk diluted in tris-buffered saline/Tween (TBST), the membranes were washed with TBST and then incubated with primary antibodies overnight at 4°C. The following antibodies were used for signaling pathway analysis: rabbit anti-Collagen I (Abcam, ab270993), rabbit anti-Collagen III (Abcam, ab184993), rabbit anti-alpha smooth muscle actin (Abcam, ab124964), and mouse anti-GAPDH (Beijing Ray Antibody Biotech, RM2002). After washing, the membranes were incubated with horseradish peroxidase (HRP)-conjugated secondary antibodies [Goat anti-Mouse IgG (Beijing Ray Antibody Biotech, RM3001); Goat Anti-Rabbit IgG (Beijing Ray Antibody Biotech, RM3002)] for 1 h at 37°C. Bound antibodies were detected using the ECL Western blotting detection system.

### 2.11 Statistical analysis

All experiments were performed in triplicate and repeated at least three times. Statistical analyses were performed using SPSS software, version 19.0 (IBM, Armonk, NY, USA). Data represent mean ± standard deviation. A two-tailed, unpaired Student’s t-test or the Mann-Whitney U test was employed to compare the values between subgroups for quantitative data. P < 0.05 was considered to be statistically significant.

## 3 Results

### 3.1 Single-cell RNA-seq reveals immune cell heterogeneity of fibrotic skin diseases and normal scar dermis tissues

**Figure 1:**
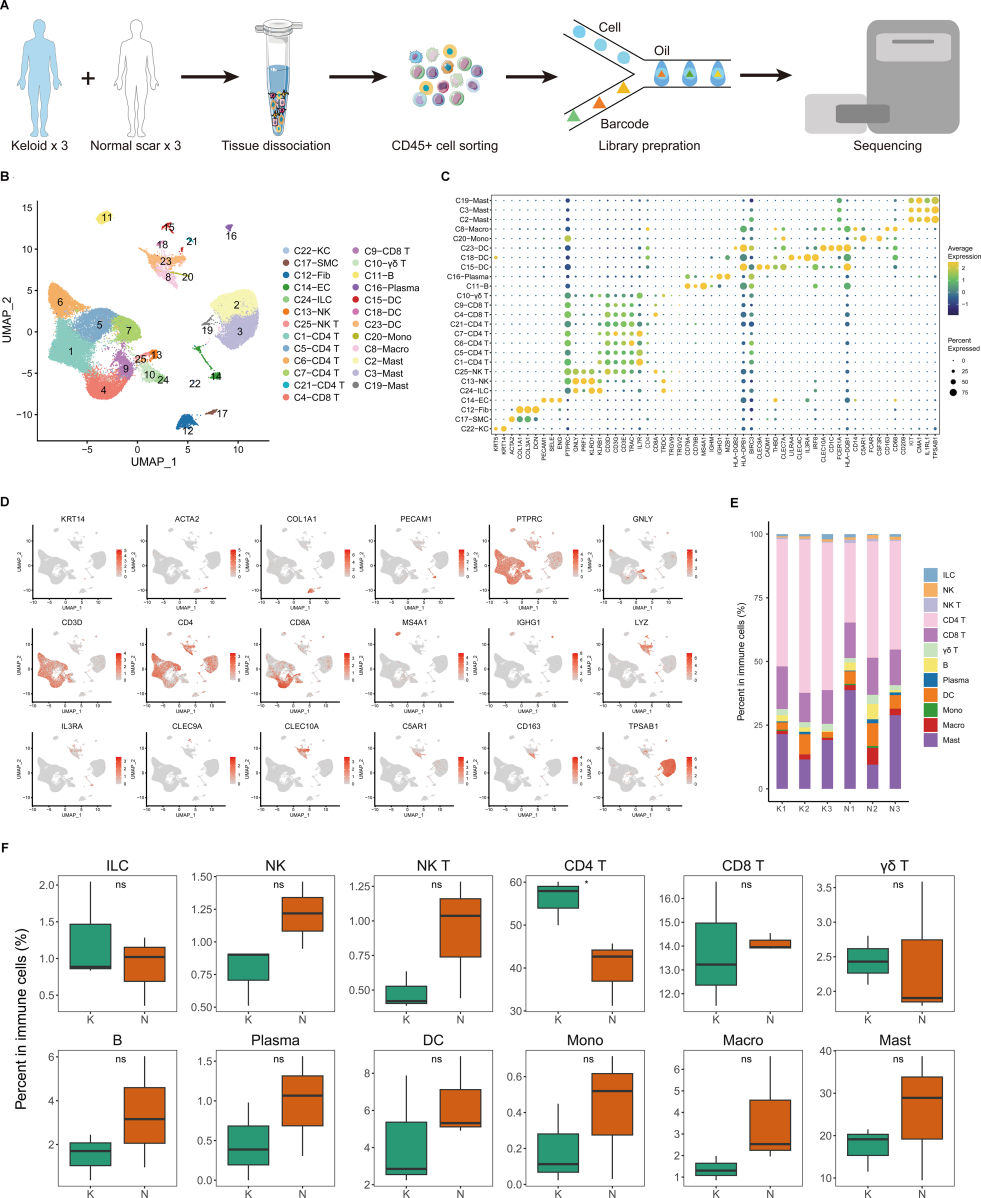
Single-cell transcriptome map of immune cells in fibrotic skin disease and normal scar dermis samples. **(A)** Workflow depicting the collection and processing of keloid, a paradigm of fibrotic skin diseases, and normal scar CD45^+^ cells for scRNA-seq. **(B)** Unsupervised clustering of the 41,084 single cells from three keloid samples and three normal scar samples, including 25 clusters and 16 major clusters. KC, keratinocyte; SMC, smooth muscle cell; Fib, fibroblast; EC, endothelial cell; ILC, innate lymphoid cell; NK, natural killer; DC, dendritic cell; Mono, monocyte; Macro, macrophage. **(C)** Dot plot of the expression of key cell type marker genes in each cell cluster. Bubble size is proportional to the percentage of cells expressing a gene in a cluster and color intensity is related to the average scaled gene expression. **(D)** Feature plots of expression distribution for cell type-specific markers. **(E)** The proportion of each cell type in three keloid samples and three normal scar samples. K, keloid; N, normal scar. **(F)** The percentage of cells for each immune cell type in keloids and normal scars. Ns, not significant; *, *P*<0.05; K, keloid; N, normal scar.

To explore the immunological profile of fibrotic skin disease, we used FACS to isolate CD45^+^ cells from keloid, a paradigm of fibrotic skin diseases, and normal scar dermis tissues for scRNA-seq (**Figure 1A**). We chose CD45 to enrich immune cells because CD45 has been suggested to express on almost all hematopoietic cells except for mature erythrocytes (25–27). We only used the dermis for scRNA-seq analysis because keloid is a skin dermis fibrotic disease. After stringent quality control (**Supplementary Figures S1A, B**), we obtained the transcriptomes of 41,084 cells. Unsupervised UMAP clustering revealed 25 cell clusters (**Figure 1B**; **Supplementary Figure S1C**). Based on established lineage-specific marker genes (**Figures 1C, D**; **Supplementary Figure S1D**), we assigned these clusters to multiple cell lineages. The immune cell lineage was identified by PTPRC (**Figure 1D**). T cells, macrophages, dendritic cells (DCs), and mast cells accounted for the majority of the sequenced cells. Some cells expressed non-immune cell markers, such as fibroblast or endothelial cell markers, which may have resulted from the incomplete removal of these cells by FACS.

We next analyzed the proportions of immune cell lineages in keloids and normal scars. The immune cell lineages in the dermis of keloids and normal scars showed distinct relative cell number ratios (**Figures 1E, F**). The proportion of CD4^+^ T cells increased significantly in keloids compared to normal scars, suggesting that CD4^+^ T cells may play an important role in keloid development (**Figure 1F**). The proportions of natural killer (NK) cells, NK T cells, B cells, and macrophages were decreased in keloid tissues compared to normal scar tissues, although the difference is not significant. Some other T cells, such as CD8 T cells and γδ T cells, showed similar proportions in keloids and normal scars (**Figure 1F**).

### 3.2 T cell subclustering into distinct cell populations and Th17 cells are increased in fibrotic skin disease

**Figure 2:**
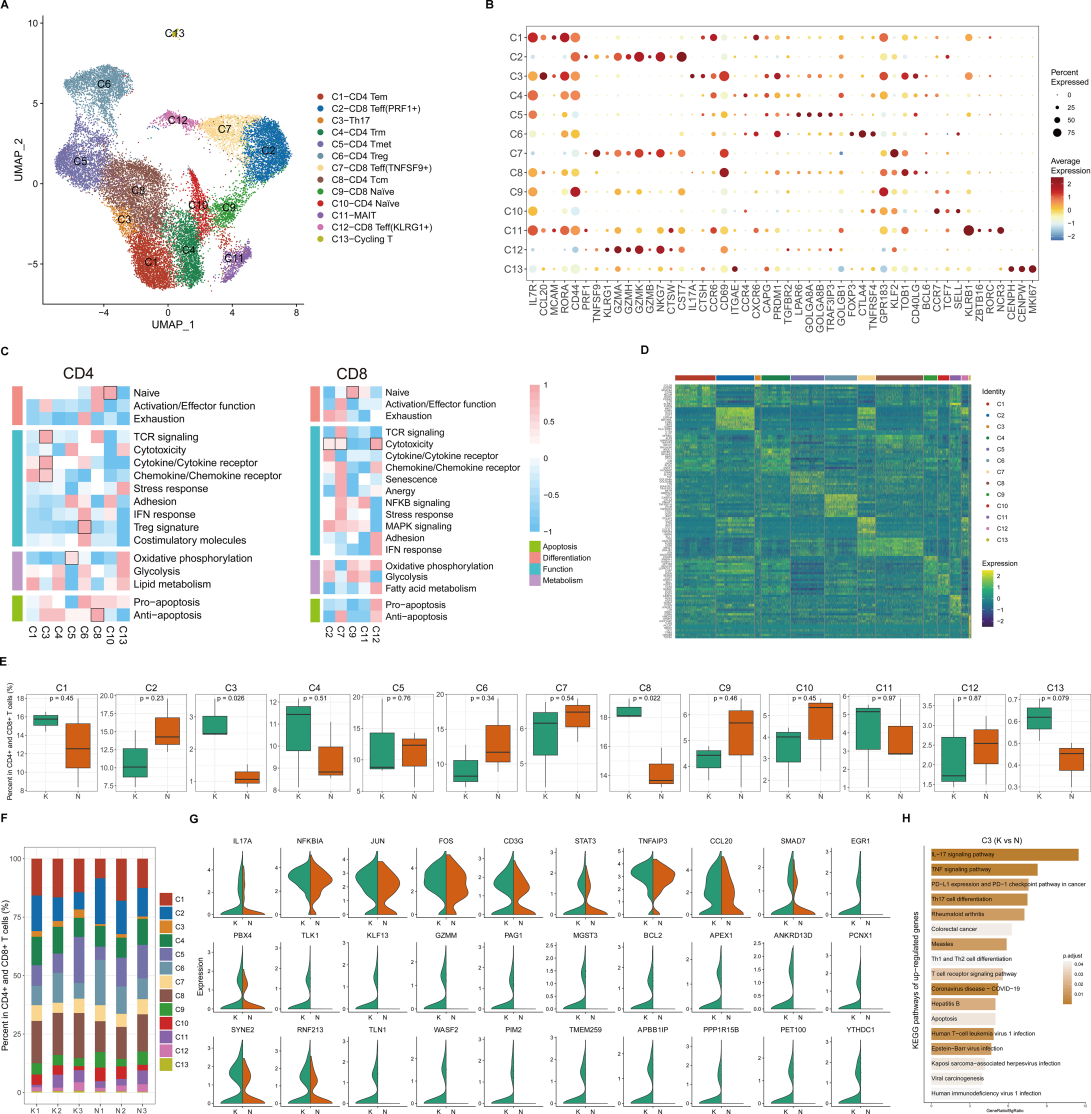
Transcriptional diversity of CD4^+^ and CD8^+^ T cells. **(A)** Uniform Manifold Approximation and Projection (UMAP) of 13 subclusters identified in CD4^+^ and CD8^+^ T cells. **(B)** Dot plots showing distinct expressions of the selected marker genes in each subcluster. **(C)** Heatmap illustrating the scaled score calculated based on the expression of curated gene signatures across CD4^+^ T cell subclusters (left) and CD8^+^ T cell subclusters (right). **(D)** Heatmap of the top 10 differentially expressed genes (ranked by log-transformed fold change in descending order) in the CD4^+^ and CD8^+^ T cell subclusters. **(E)** Box plots showing the percentage of cells for each T cell subcluster in the keloid and normal scar samples. The p-value indicated in the plot was calculated by unpaired two-tailed t-tests. **(F)** The proportion of each T cell subcluster in three keloid samples and three normal scar samples. K, keloid; N, normal scar. **(G)** Violin plots showing differentially expressed genes in the Th17 cells in keloids and normal scars. K, keloid; N, normal scar. **(H)** Functional KEGG pathway enrichment of the upregulated genes (keloid vs. normal scar, avg_logFC > 0.25, and p value < 0.05) in Th17 cells. The p-value was calculated using the hypergeometric distribution and corrected using the Benjamini and Hochberg method. Pathways with an adjusted p-value of <0.05 are considered significant.

Because CD4^+^ T cells undergo significant changes in keloids compared to normal scars (**Figure 1F**), and T cells are important for keloid pathogenesis, we next performed unsupervised clustering of all keloid and normal scar T cells (**Figure 2A**; **Supplementary Figure S2A**). Based on DEGs, canonical immune markers, and curated gene signatures (**Figures 2B–D**; **Supplementary Figure S2B**), we defined 13 transcriptional states: naïve T (C9 and C10), CD8 Teff (C2, C7, and C12), Th17(C3), CD4 Trm (C4), CD4 Tmet (C5), CD4 Treg (C6), CD4 Tcm (C8), MAIT (C11), and Cycling T (C13) (**Figures 2A–D**). **Figures 2E, F** show the cell proportions of the T cell subclusters in keloids and normal scars. From the results, we can see that the proportion of Th17 cells and CD4 Tcm cells was consistently increased in the keloid samples compared to the normal scar samples (**Figures 2E, F**).

Because Th17 cells play an important role in the pathogenesis of a diverse group of inflammation-mediated skin diseases, and inflammation is important for keloid pathogenesis, we next focused on Th17 cells. We compared differences between keloid Th17 cells and normal scar Th17 cells. We identified genes associated with the IL-17 and TNF signaling pathways, such as IL-17A, TNFAIP3, and CCL20, which were significantly increased in keloid Th17 cells (**Figure 2G**). KEGG pathway analysis also suggested that the IL-17 signaling pathway, TNF signaling pathway, and Th17 cell differentiation-associated pathway were enriched in the keloid Th17 cells (**Figure 2H**; **Supplementary Figure S2C**). These results suggest that not only was the proportion of Th17 cells increased but also the identities of Th17 cells changed in keloids compared to normal scars.

We next integrated the scRNA-seq data from CD45^+^ cells in healthy skin tissues (26) into our study. We integrated and analyzed 73,597 single cells from three keloid, three normal scar, and seven healthy control skin samples (**Supplementary Figures S3A, B**). The results also showed that the proportion of CD4^+^ T cells increased significantly in keloids compared to normal scars and healthy skin (**Supplementary Figure S3C**). We next performed unsupervised clustering of all T cells in the keloid, normal scar, and healthy skin samples. Based on canonical immune markers and curated gene signatures, we defined 15 subclusters (**Supplementary Figures S3D–F**). The proportion of Th17 cells was increased in keloids compared to healthy skin (**Supplementary Figure S3G**), which is consistent with the finding in normal scars. We also found that the proportion of CD8 Teff (IFNG+) was consistently increased in keloids and normal scars compared to healthy skin (**Supplementary Figure S3G**), suggesting that the cells may play a role in scar formation. We next compared differences in the keloid Th17 cells, normal scar Th17 cells, and healthy skin Th17 cells. IL-17A, TNFAIP3, and CCL20 were found to be significantly increased in the keloid Th17 cells compared to the healthy skin Th17 cells (**Supplementary Figure S3H**). We also found that the Th17-type immune response and IL17-mediated signaling pathway were enriched in the keloid Th17 cells compared to the healthy skin Th17 cells (**Supplementary Figure S3I**).

We also analyzed Tregs, another important cell in immune regulation. We performed unsupervised clustering on all Tregs in keloids and normal scars. We observed further heterogeneity with two subclusters, KLF2^+^ Tregs and LAIR2^+^ Tregs (**Supplementary Figure S4A**). Pathway analysis suggested that the upregulated genes in the LAIR2^+^ Tregs were associated with a response to the interleukin-2 and interleukin-15-mediated signaling pathway and the upregulated genes in the KLF2^+^ Tregs were associated with the regulation of protein stability and fibrillar center (**Supplementary Figure S4B**). Both Treg subclusters showed similar proportions in the keloid and normal scar samples (**Supplementary Figure S4C**). We next compared the differences between the keloid Tregs and normal scar Tregs. GO analysis showed that the upregulated genes in the keloid KLF2^+^ Tregs were associated with the platelet-derived growth factor receptor signaling pathway and the regulation of B cell activation, and the upregulated genes in the keloid LAIR2^+^ Tregs were associated with the toll-like receptor 2 signaling pathway (**Supplementary Figure S4D**).

### 3.3 Transcriptional landscapes reveal the heterogeneity of mono-macrophages and increased macrophage activity in fibrotic skin disease

**Figure 3:**
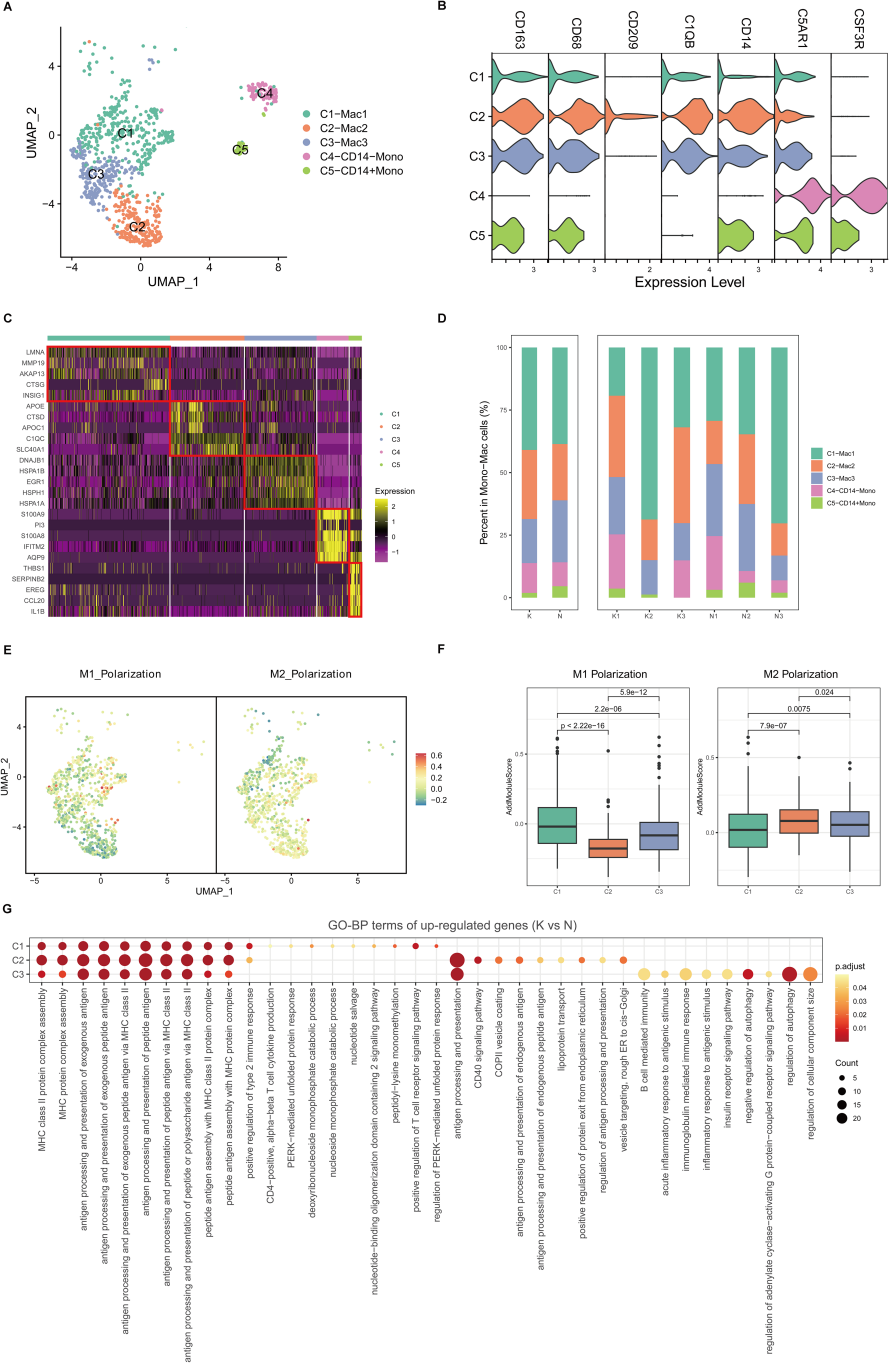
Fibrotic skin disease and normal scar mono-macrophages subclustered into distinct cell populations. **(A)** Uniform Manifold Approximation and Projection (UMAP) plots of the mono-macrophage subpopulations. **(B)** Violin plot showing key marker gene expression between the mono-macrophage subpopulations. **(C)** Heatmap showing the expression of the top 5 differentially expressed genes in the mono-macrophage subpopulations. **(D)** Bar plots showing the percentage of each mono-macrophage subpopulation in the keloid and normal scar samples. **(E)** UMAP plots showing the M1 and M2 scores for each cell in the macrophages. **(F)** Box plots showing the M1 and M2 scores for each subpopulation of macrophages. Significance was determined by the unpaired two-tailed t test. **(G)** GO terms enrichment of the upregulated genes (keloid vs. normal scar, avg_logFC > 0.25, and p-value < 0.05) in macrophages. GO terms with an adjusted p-value of <0.05 are considered significant.

Because mono-macrophages are reported to play an important role in fibrotic skin disease pathogenesis (9, 11), we next performed unsupervised clustering of all mono-macrophages. Based on DEGs and canonical mono-macrophage markers, we observed further heterogeneity with five subclusters, C1 through C5 (**Figures 3A–C**). C1, C2, and C3 were macrophages, and C4 and C5 were monocytes. All the mono-macrophage subclusters expressed canonical CD14, CD68, and CD163 except the C4 subcluster, which highly expressed C5AR1 and CSF3R. **Figure 3D** shows the cell proportions of the mono-macrophage subclusters in keloids and normal scars. From the results, we can see that all five subclusters showed similar proportions in the keloid samples and normal scar samples.

Macrophages can be divided into M1 macrophages and M2 macrophages (28). By calculating M1 and M2 polarization scores using related gene sets, we found that the C1 macrophages were more like M1 macrophages, and the C2 macrophages were more like M2 macrophages. The C3 macrophages were like the intermediate state between M1 and M2 macrophages (**Figures 3E, F**). We next compared differences between keloid mono-macrophages and normal scar mono-macrophages (**Figure 3G**; **Supplementary Figures S5A–C**). GO analysis showed that the upregulated genes in the keloid C1, C2, and C3 macrophages compared to the normal scar macrophages were all associated with MHC class II protein complex assembly and antigen processing and presentation (**Figure 3G**), suggesting the consistent active state of macrophages in keloids.

### 3.4 Transcriptional landscapes reveal heterogeneity of dendritic cells and increased cDC2 and migDC cell activity in fibrotic skin disease

**Figure 4:**
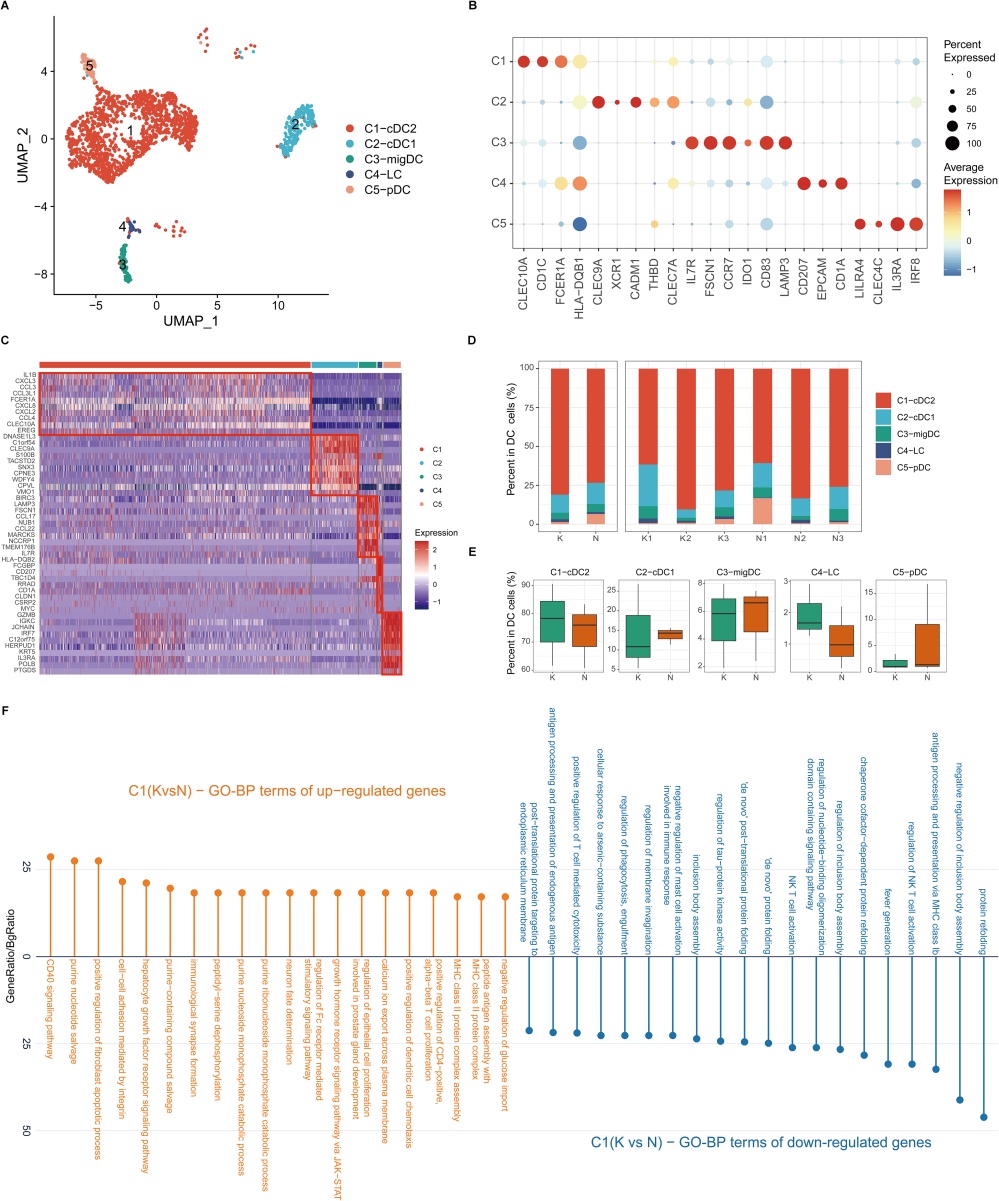
Fibrotic skin disease and normal scar dendritic cells (DCs) subclustered into distinct cell populations. **(A)** Uniform Manifold Approximation and Projection (UMAP) plot showing the annotation and color codes for subclusters of DCs. **(B)** Dot plot of representative genes of cell types in DCs. **(C)** Heatmap showing the expression of the top 10 differentially expressed genes in each subcluster of DCs. **(D)** Percentage distribution of each subcluster in the keloid and normal scar samples. **(E)** Box plots showing the percentage of DC subclusters in keloids and normal scars. **(F)** GO terms enrichment of differentially expressed genes in cDC2 cells. GO terms with an adjusted p-value of <0.05 are considered significant.

DCs are important antigen-presenting cells (APCs) in the skin. We next performed unsupervised clustering of all DCs in keloids and normal scars. Consistent with previous reports, DCs in the skin were clustered into cDC1, cDC2, pDC, migDC, and Langerhans cells (LCs) (**Figures 4A–C**). Most of the DCs in keloids and normal scars were cDC2 (**Figures 4A–C**). There were several LCs in the results, which may have resulted from the incomplete removal of the epidermis. Cell proportion analysis suggested that the five subclusters showed similar proportions in the keloid and normal scar samples (**Figures 4D, E**).

We next compared differentially expressed genes in the keloid DCs and normal scar DCs (**Figure 4F**; **Supplementary Figures S6A–D**). GO analysis showed that the upregulated genes in keloid cDC2 and migDC were associated with MHC class II protein complex assembly and peptide antigen assembly (**Figure 4F**; **Supplementary Figure S6B**), suggesting the active state of cDC2 and migDC in keloids.

### 3.5 Transcriptional landscapes reveal heterogeneity of mast cells in keloids and increased IL-17 signaling in mast cells in fibrotic skin disease

**Figure 5:**
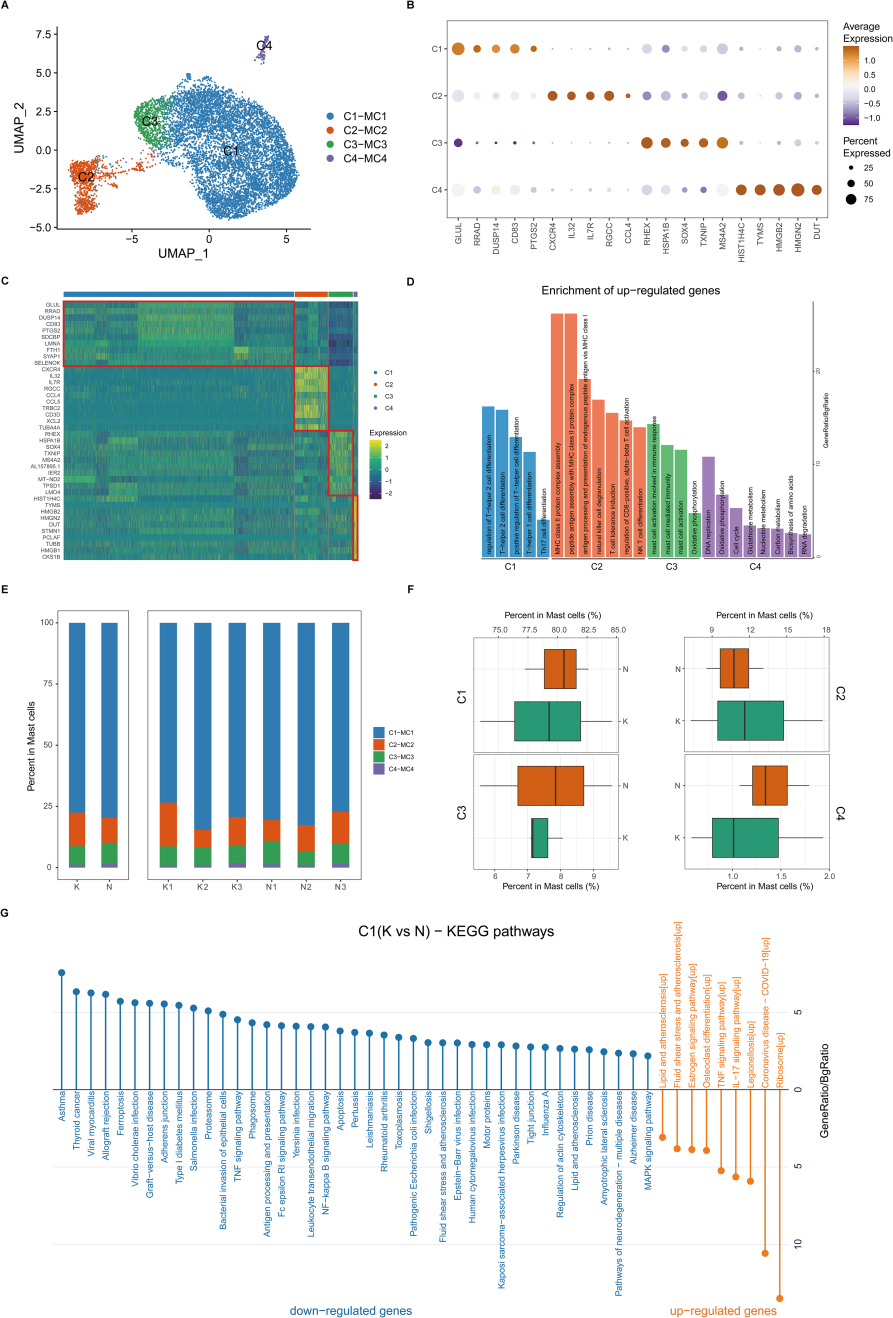
Fibrotic skin disease and normal scar mast cells subcluster into distinct cell populations. **(A)** Uniform Manifold Approximation and Projections (UMAPs) of subclustered mast cells, labeled in different colors. Cell type annotations are provided in the figure. **(B)** Dot plot indicating the expression of selected gene sets in mast subclusters. **(C)** Scaled expression of differentially expressed genes in mast subclusters. **(D)** Enrichment of differentially expressed genes in one mast subcluster compared to other mast subclusters. Results with adjusted P-value of <0.05 are considered significant. **(E)** Bar plot showing the fraction of mast subcluster in keloid and normal scar samples. **(F)** Boxplot showing the fraction of mast subclusters in keloid and normal scar. **(G)** KEGG pathway enrichment of differentially expressed genes in C1 subcluster. GO terms with adjusted P-value of <0.05 are considered significant.

Mast cells are reported to play an important role in fibrotic skin disease pathogenesis (7, 9, 11). We next performed unsupervised clustering of all mast cells in keloids and normal scars (**Figure 5A**). Based on differentially expressed genes (**Figures 5B–D**), we observed further heterogeneity with four subclusters, C1 through C4 (**Figures 5A–D**). The C1 subcluster constitutes the majority of the mast cells and highly expressed GLUL, RRAD, DUSP14, and so on. **Figures 5E** and **F** show the cell proportions of the mast cell subclusters in keloids and normal scars. From the results, we can see that all four subclusters showed similar proportions in the keloid and normal scar samples.

We next compared differentially expressed genes in the keloid mast cells and normal scar mast cells (**Figure 5G**; **Supplementary Figures S7A–D**). KEGG analysis showed that the upregulated genes in the keloid C1 and C2 mast cell subclusters were associated with the IL-17 signaling pathway (**Figure 5G**; **Supplementary Figure S7A**), suggesting that activating IL-17 signaling in the keloid microenvironment may act on mast cells.

### 3.6 Transcriptional landscapes reveal heterogeneity of B cells and increased plasma cell activity in fibrotic skin disease

Like T cells, B cells are important lymphocytes in the immune system. We next performed unsupervised clustering of all B cells in keloids and normal scars (**Supplementary Figure S8A**). Consistent with previous reports, B cells in the skin can be clustered into naïve B cells, activated B cells, and plasma cells (**Supplementary Figure S8A**). A dot plot shows the expression of specific markers in the B cell subpopulations (**Supplementary Figure S8B**). Cell proportion analysis suggested that the three subclusters showed similar proportions in the keloid and normal scar samples (**Supplementary Figure S8C**).

We next compared differentially expressed genes in keloid B cells and normal scar B cells (**Supplementary Figure S8D**). GO analysis showed that the upregulated genes in the keloid plasma cells were associated with antigen binding, the immunoglobulin complex, and the MHC protein complex (**Supplementary Figure S8D**), suggesting the active state of plasma cells in keloids.

### 3.7 Potential ligand–receptor interactions in fibrotic skin disease and normal scars

**Figure 6:**
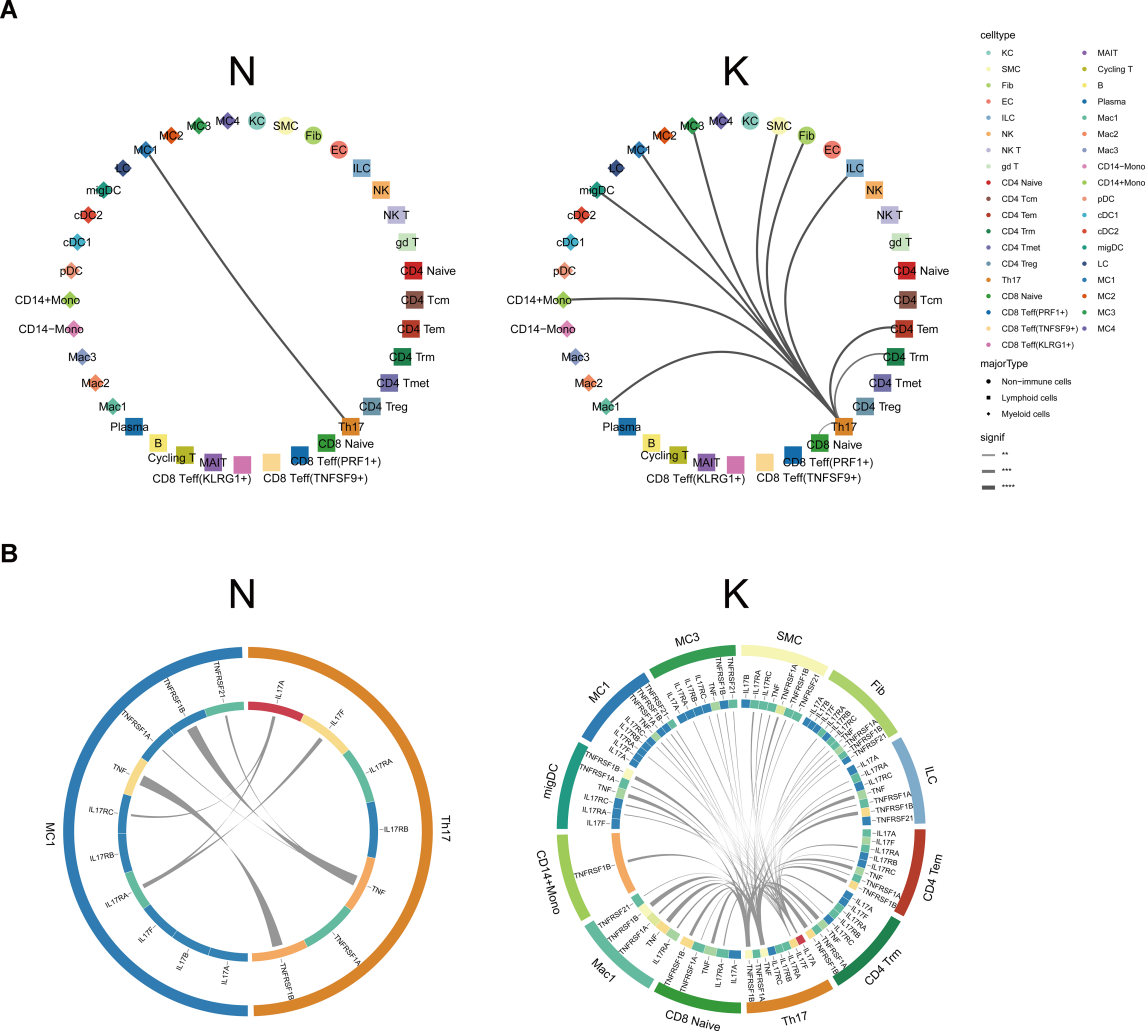
Cellular interactions between Th17 cells and other cell types. **(A)** CSOmap analysis showing the interaction between Th17 cells and other cell subsets in normal scars (left) and keloids (right). Line thickness represents the significance of the cell-cell interaction. **(B)** Putative ligand and receptor pairs related to IL-17 and TNF within the Th17 cells and other cell populations in normal scars (left) and in keloids (right). The color of the middle ring is related to the average expression of genes in the cell types, with red representing a high expression and blue representing a low expression. The thicker the line, the greater the contribution of the ligand-receptor pairs. **, p<0.01, ***, p<0.001, ****, p<0.0001.

The single-cell dataset provided us with a unique chance to analyze cell-cell communication mediated by ligand-receptor interactions. To define the cell-cell communication landscape in keloids and normal scar immune cells, we used CSOmap, a bioinformatics tool to infer the spatial organization of tissues and molecular determinants of cellular interaction (29). We observed a significant increase in cell-cell communications in keloids compared to normal scars (**Supplementary Figure S9A**). Interestingly, the cell-cell communications between Th17 cells and other cells increased significantly in keloids compared to normal scars (**Figure 6A**), suggesting the active cell communication of Th17 cells and its important role in keloids. The main ligand-receptor pairs contributing to the cell-cell communications between Th17 cells and other cells were IL-17A, IL-17F, TNFα, and their receptors (**Figure 6B**).

### 3.8 Th17 cells promote proliferation, collagen expression, and migration of fibrotic skin disease fibroblasts by secreting IL-17A

**Figure 7:**
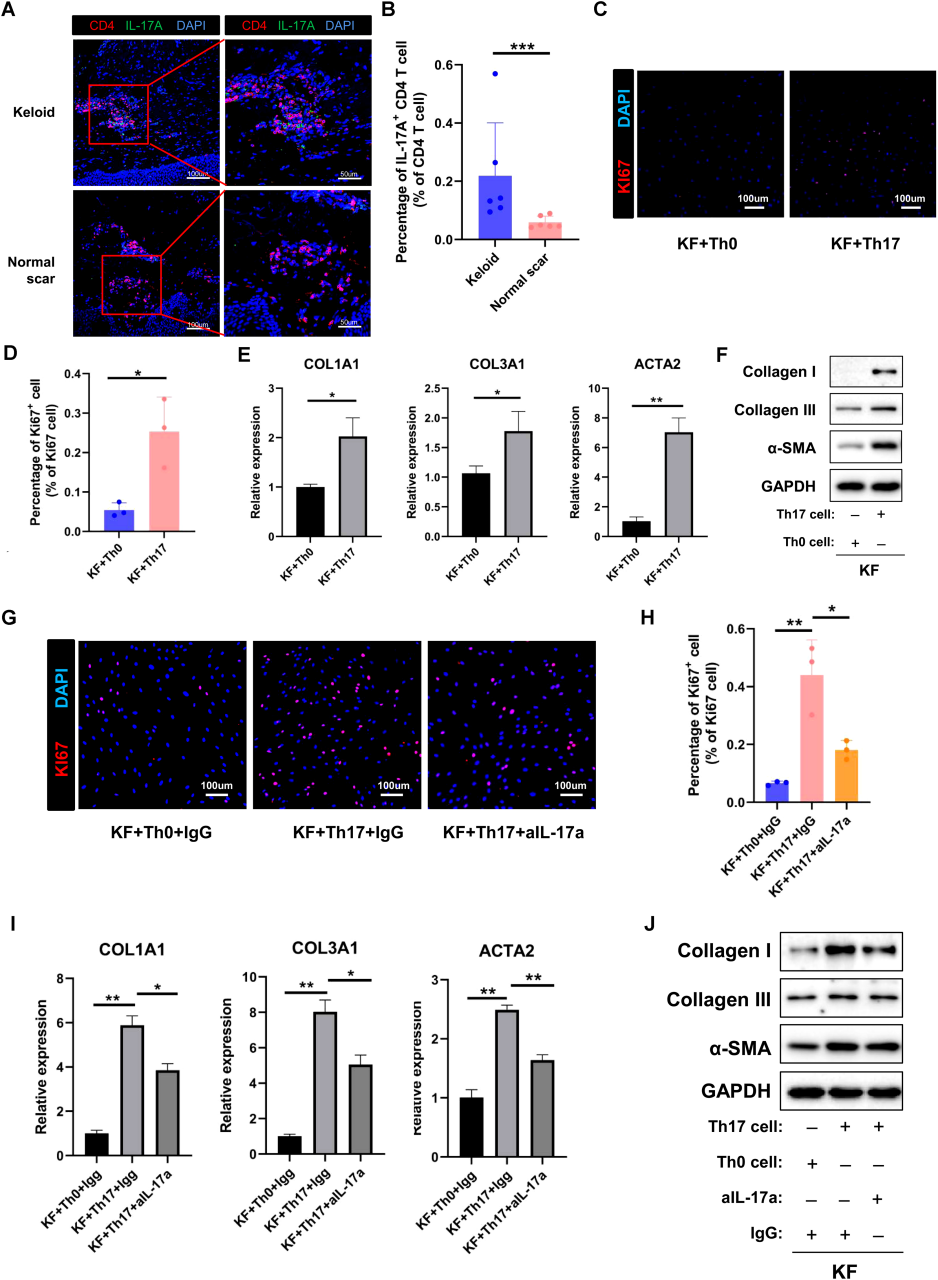
Th17 cell promotes the proliferation and collagen expression of keloid fibroblasts by secreting IL-17A. **(A)** Immunofluorescence staining of IL-17A and CD4 in keloid and normal scar tissues. The right panels are the insets of the left panels. Scale bar = 100 μm (left panel) and 50 μm (right panel). **(B)** Percentage of IL-17A^+^/CD4^+^ cells in normal and keloid tissues. Error bars represent SD (n=6). ***, *p*<0.001. **(C, D)** Ki67 staining analysis of fibroblasts co-cultured with Th0 or Th17 cells. Scale bar = 100 μm. Error bars represent SD (n=3). *, *P*<0.05. **(E, F)** qRT-PCR and Western blot analysis of collagen I, collagen III, and α-SMA expression in fibroblast co-cultured with Th0 or Th17 cells. Error bars represent SD (n=3). *, *p*<0.05; **, *P*<0.01. **(G, H)** Ki67 staining analysis of fibroblasts co-cultured with Th0 or Th17 cells in the presence or absence of anti-IL-17A antibody. Error bars represent SD (n=3). *, *p*<0.05; **, *p*<0.01. **(I, J)** qRT-PCR and Western blot analysis of collagen I, collagen III, and α-SMA expression in fibroblasts co-cultured with Th0 or Th17 cells in the presence or absence of anti-IL-17A antibody. Error bars represent SD (n=3). *p<0.05; **, p<0.01.

Based on the scRNA-seq analysis, the percentage of Th17 cells was significantly increased in keloids compared to normal scars. To validate this finding, we performed immunofluorescence (IF) staining on skin tissues derived from normal controls and keloids. Th17 cells were identified based on CD4 and IL-17A expression (**Figure 7A**). The IF staining results showed that the proportion of IL-17A^+^/CD4^+^ cells was higher in keloids than in the normal controls (**Figures 7A, B**). This result is consistent with the scRNA-seq transcriptomics analysis. To explore the function of Th17 cells in keloids, we induced Th17 cells from keloid patients *in vitro* (**Supplementary Figure S10A**) and subsequently co-cultured them with primary keloid fibroblasts (KF) isolated from keloid patients. After co-culturing with Th17 cells, the KFs exhibited a significant increase in collagen I/III and α-SMA expression and in their proliferative and migratory capabilities, compared to the control groups (**Figures 7C–F**; **Supplementary Figures S10B, C**). IL-17A has been reported to be the key molecule for Th17 cells’ functions in fibrotic diseases. To ascertain whether the increased collagen I/III expression and proliferative and migratory capabilities of KFs had resulted from IL-17A secreted by Th17 cells, we next introduced a neutralizing antibody against IL-17A into the co-culture system of Th17 cells and KFs. The IL-17A neutralizing antibody inhibited the increased expression of collagen I and III and the proliferative and migratory capabilities of the KFs co-cultured with Th17 (**Figures 7G–J**; **Supplementary Figures S10D, E**), suggesting that Th17 cells promote proliferation, collagen expression, and migration of keloid fibroblasts through IL-17A.

### 3.9 Th17 cells are increased in hypertrophic scars and scleroderma

**Figure 8:**
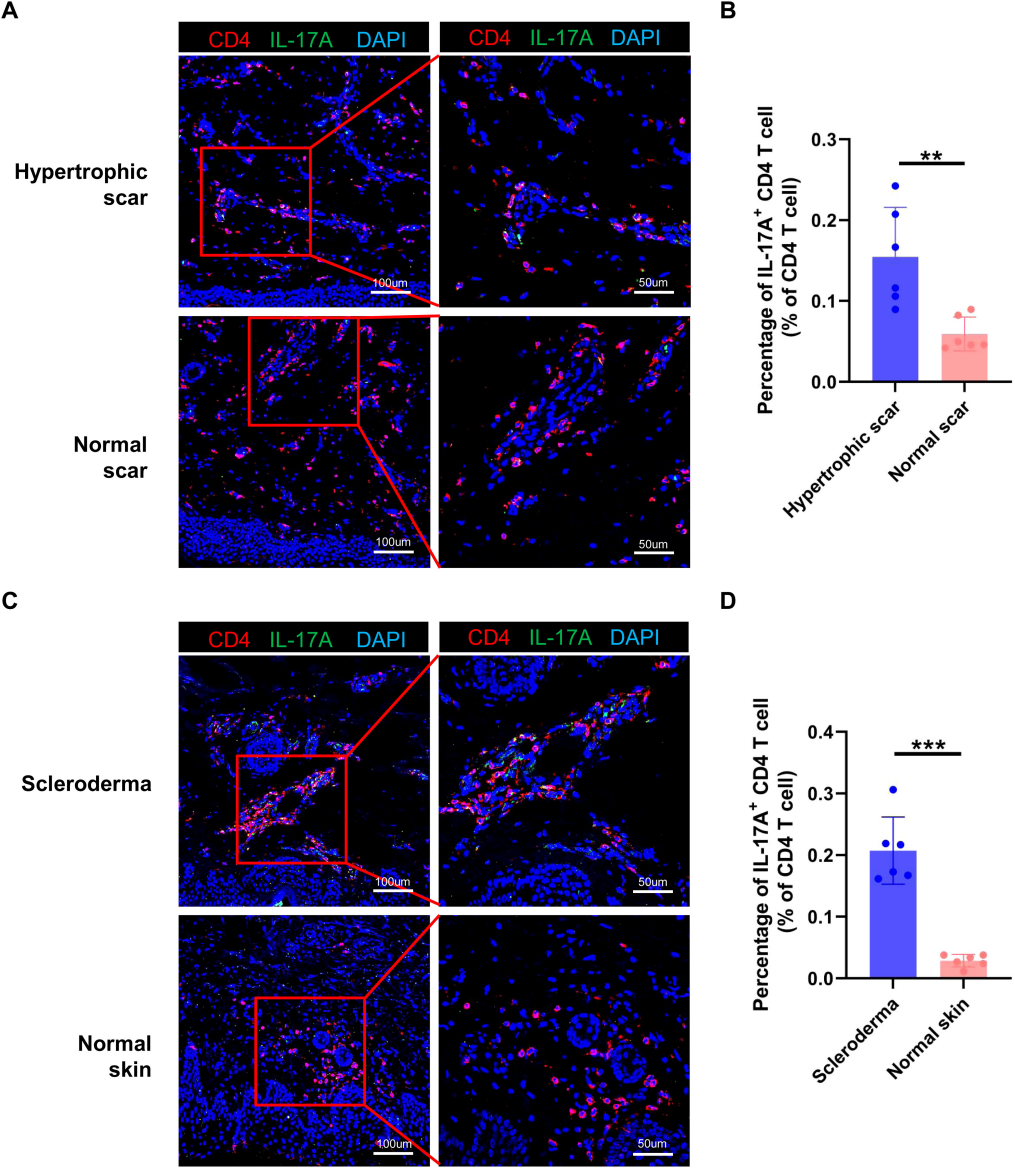
Th17 cells are increased in hypertrophic scars and scleroderma. **(A)** Immunofluorescence staining of IL-17A and CD4 in hypertrophic scar and normal scar tissues. The right panels are the insets of the left panels. Scale bar = 100 μm (left panel) and 50 μm (right panel). **(B)** Percentage of IL-17A^+^/CD4^+^ cells in hypertrophic scar and normal scar tissues. Error bars represent SD (n=6). **, *p*<0.01. **(C)** Immunofluorescence staining of IL-17A and CD4 in scleroderma and normal skin tissues. The right panels are the insets of the left panels. Scale bar = 100 μm (left panel) and 50 μm (right panel). **(D)** Percentage of IL-17A^+^/CD4^+^ cells in scleroderma and normal skin tissues. Error bars represent SD (n=6). ***, *p*<0.001.

To examine the consistency of our findings in other fibrotic skin diseases, we performed immunofluorescence staining in hypertrophic scar and scleroderma tissues. The immunofluorescence staining results showed that the proportion of IL-17A^+^/CD4^+^ cells was higher in hypertrophic scar and scleroderma tissues than in normal control tissues (**Figures 8A–D**). Taken together, these results indicated that increasing Th17 cells may be a universal mechanism in fibrotic skin diseases.

## 4 Discussion

Immune cells and inflammation have been reported to be important for the pathogenesis of fibrotic skin diseases (7, 9, 11). Although there have been some studies exploring the composition of immune cells in fibrotic skin diseases, these studies detected the gene expression of all cells in fibrotic skin disease tissues and did not enrich immune cells (13–15). In this study, we built a single-cell atlas of fibrotic skin disease and normal scar immune cells using FACS-enriched CD45^+^ cells and explored the function of the Th17 cell-fibroblast interaction in the pathogenesis of fibrotic skin disease. These findings will help us understand fibrotic skin disease pathogenesis in depth, and provide potential targets for clinical therapies for fibrotic skin diseases.

Mast cells, Treg cells, and M2 macrophages have been reported to play important roles in the pathogenesis of fibrotic skin disease (6, 7, 11, 12). These cells have been suggested to be increased and promote extracellular matrix deposition in keloid tissues. However, in our single-cell atlas of keloid and normal scar immune cell research, we found that there were no differences in the proportions of these cells in keloids and normal scars. The gene expression of these cells had significant differences in keloids and normal scars. The upregulated genes in the keloid C1 mast cell subcluster, the major subcluster of mast cells, were associated with lipids and atherosclerosis, the TNF signaling pathway, and the IL-17 signaling pathway (**Figure 5G**). The upregulated genes in the keloid Treg cells, compared to normal scar Treg cells, were associated with the TNF signaling pathway, IL-17 signaling pathway, and apoptosis (**Supplementary Figure S2C**). The upregulated genes in the keloid M2 macrophages, compared to normal scar M2 macrophages, were associated with MHC class II protein complex assembly and antigen processing and presentation (**Figure 3G**). These findings suggested that not the change in cell proportions but the change in gene expression of mast cells, Treg cells, and M2 macrophages may contribute to fibrotic skin disease development.

Th17 cells are key cells for host protection against mucosal infections and are major pathogenic cells in multiple autoimmune and inflammatory diseases, including psoriasis and systemic lupus erythematosus (16, 30, 31). IL-17 has been reported to be the major effector molecule of Th17 in the aforementioned functions (32, 33). In recent years, the roles of Th17 cells in fibrotic diseases have been paid increasing attention. Th17 cells have been reported to play important roles in intestinal fibrosis, lung fibrosis, and myocardial fibrosis (18, 34, 35). However, the functions of Th17 cells in keloids are still unknown. In this study, we found that the percentage of Th17 cells was significantly increased in keloids compared to normal scars and Th17 cells promoted the collagen expression, proliferation, and migration of keloid fibroblast (**Figures 2E**, **7**, **Supplementary Figure S10**). Mechanism studies showed that the Th17 cells performed the above functions by secreting IL-17A (**Figure 7**, **Supplementary Figure S10**), which is consistent with previous findings (36, 37). Importantly, we also found an increased number of Th17 cells in hypertrophic scars and scleroderma compared to normal controls (**Figure 8**). These results suggested that Th17 cells may have an important role in multiple skin fibrosis diseases, and may serve as target cells for fibrosis treatment.

The mechanism that IL-17A promotes fibrotic diseases is complex, organ-specific, and disease-specific. IL-17A has been reported to promote the fibrosis of systemic sclerosis by increasing inflammation and the proliferation and collagen deposition of fibroblasts (38, 39). The increased level of IL-17A in liver fibrosis facilitates the influx of inflammatory cells, drives the expression of profibrogenic growth factors, and activates hepatic stellate cells in the liver (40–42). The liver-infiltrating inflammatory cells in turn induce the production of profibrotic cytokines such as TNF-α, IL-6, IL-1, and TGF-β1 to accelerate fibrosis (40). Several studies have suggested that IL-17 directly interacts with colonic IL-17R, expressing myofibroblasts and contributing significantly to stricture development in Crohn’s disease (43–45). IL-6, IL-8, and MCP-1 secretions were rapidly induced by IL-17 in colonic subepithelial myofibroblasts (43). *In vitro* stimulation of IL-17 induced HSP47 and type I collagen in human intestinal myofibroblasts (45). The mechanism by which IL-17A facilitates fibrosis in keloids is still unclear. We will explore the mechanism using high-throughput sequencing methods and molecular biology experiments. Illustrating the downstream signaling pathways activated by IL-17A in fibroblasts of keloids can supply new targets for keloid therapy.

Both macrophages and DCs are important antigen-presenting cells in the skin. Many studies suggest that M2 macrophages are increased and play an important role in keloid development (12, 46), but the roles of DCs are still unclear in keloids. In our findings, all the subclusters of macrophages and DCs showed similar proportions between the keloid and normal scar samples (**Figures 3**, **4**). However, the upregulated genes in the keloid M1 macrophages, M2 macrophages, and cDC2 were all associated with MHC class II protein complex assembly and antigen assembly (**Figures 3G**, **4F**). These results indicate that macrophages and cDC2 are active in fibrotic skin diseases and may serve as target cells for fibrotic skin disease therapy.

In conclusion, we provided a systematic analysis of immune cell heterogeneity in fibrotic skin disease at single-cell resolution in this study. In addition, we identified that increased Th17 cells in fibrotic skin disease are involved in the proliferation, collagen expression, and migration of fibrotic skin disease fibroblasts. These findings will help us to understand fibrotic skin disease pathogenesis in depth and identify potential targets for fibrotic skin disease treatment.

## Data Availability

Single-cell RNA-seq data have been deposited in the Gene Expression Omnibus database under accession codes “GSE270438”.
