# Supplementary material for: Single-cell RNA-seq reveals immune cell heterogeneity and increased Th17 cells in human fibrotic skin diseases
Source: Front Immunol. 2025 Jan 13;15:1522076. doi: 10.3389/fimmu.2024.1522076 (PMC11769821; doi:10.3389/fimmu.2024.1522076)
Supplement: Supplementary file 1 [file DataSheet1.pdf]

Supplementary Material

Supplementary Table 1. Patient demographics and characteristics in scRNA-sequencing

No.	*Gender(F/M)*	*Age*	*Diagnosis*	*Site of Collection*
1	*F*	*20*	*Keloid*	*Chest*
2	*F*	*27*	*Keloid*	*Chest*
3	*F*	*27*	*Keloid*	*Upper arm*
4	*F*	*19*	*Normal scar*	*Upper arm*
5	*F*	*23*	*Normal scar*	*Axilla*
6	*F*	*25*	*Normal scar*	*Back*

Supplementary Table 2. Patient demographics and characteristics in immunofluorescence staining

No.	*Gender(F/M)*	*Age*	*Diagnosis*	*Site of Collection*
1	*M*	*27*	*Keloid*	*Ear*
2	*F*	*20*	*Keloid*	*Chest*
3	*F*	*19*	*Keloid*	*Chest*
4	*M*	*21*	*Keloid*	*labium*
5	*F*	*33*	*Keloid*	*Chest*
6	*M*	*26*	*Keloid*	*Ear*
7	*F*	*18*	*Normal scar*	*Face*
8	*F*	*35*	*Normal scar*	*Lip*
9	*M*	*24*	*Normal scar*	*Face*
10	*M*	*3*	*Normal scar*	*Underjaw*
11	*M*	*23*	*Normal scar*	*Back*
12	*M*	*22*	*Normal scar*	*Upper arm*
13	*F*	*26*	*Scleroderma*	*Neck*
14	*F*	*25*	*Scleroderma*	*Abdomen*
15	*F*	*11*	*Scleroderma*	*Leg*
16	*F*	*30*	*Scleroderma*	*Face*
17	*M*	*62*	*Scleroderma*	*Back*

18	*M*	*29*	*Scleroderma*	*Upper arm*
19	*F*	*29*	*Normal skin*	*Face*
20	*M*	*61*	*Normal skin*	*Abdomen*
21	*F*	*26*	*Normal skin*	*Leg*
22	*F*	*26*	*Normal skin*	*Upper arm*
23	*F*	*52*	*Normal skin*	*Back*
24	*F*	*24*	*Normal skin*	*Face*
25	*M*	*45*	*Hypertrophic scar*	*Neck*
26	*F*	*10*	*Hypertrophic scar*	*Leg*
27	*M*	*40*	*Hypertrophic scar*	*Abdomen*
28	*F*	*4*	*Hypertrophic scar*	*Chest*
29	*M*	*28*	*Hypertrophic scar*	*Upper arm*
30	*M*	*27*	*Hypertrophic scar*	*Chest*

Supplementary Table 3. The primers used in real-time PCR

Symbol	Forward primer (5'→3')	Reverse primer (5'→3')
COL1A1	TGTGCGATGACGTGATCTGTGA	CTTGGTCGGTGGGTGACTCTG
COL3A1	CAAATAGAAAGCCTCATTAGTCC	GCATCCTTGGTTAGGGTCA
ACTA2	AATAGAAGGGCTACAGCGGC	ACAGCAGCTAAGGCACCATT
GAPDH	TTGGCCAGGGGTGCTAAG	AGCCAAAAGGGTCATCATCTC

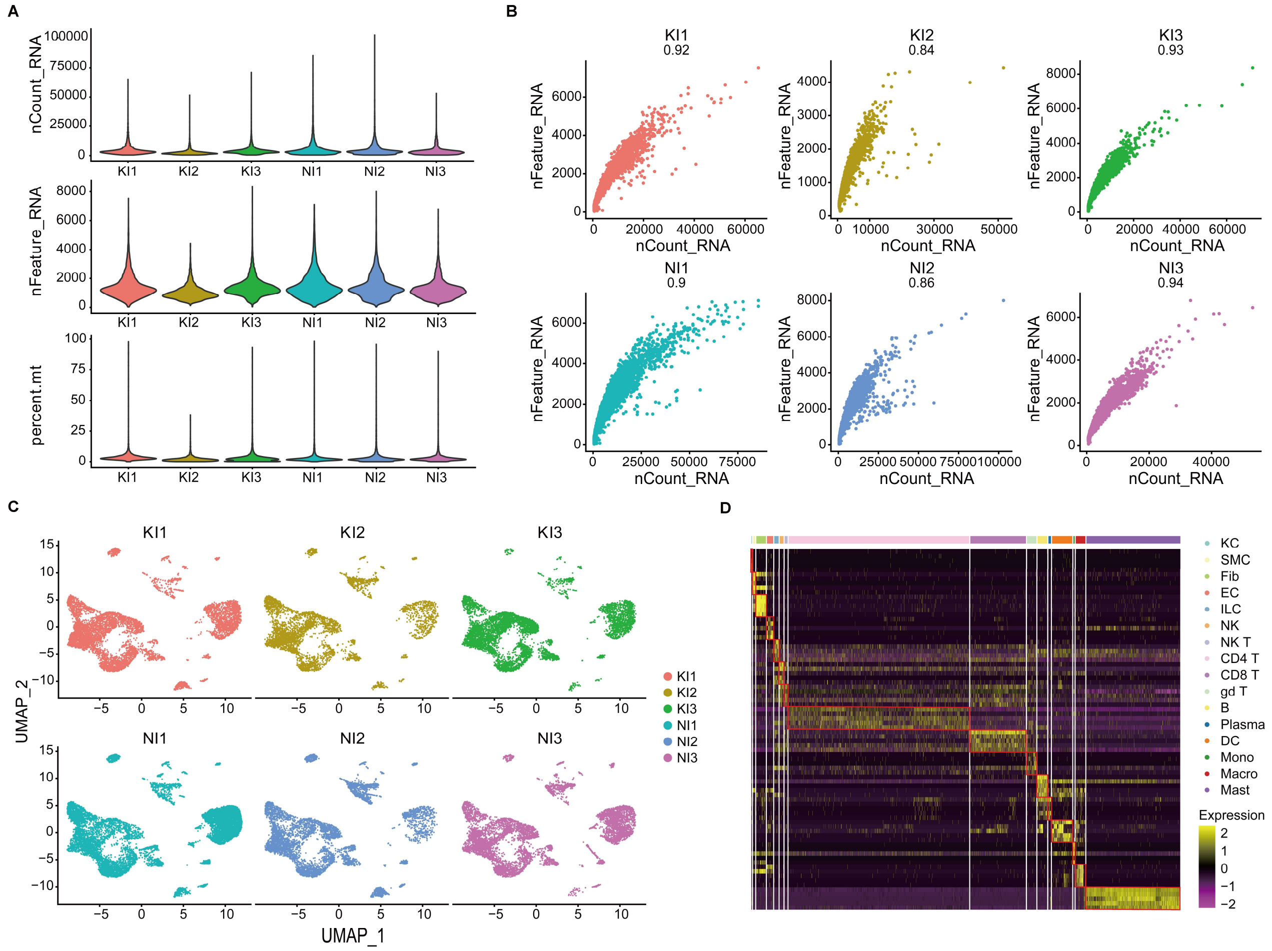

Supplementary Figure S1. Quality control of all cells from scRNA-seq of fibrotic skin disease and normal scar samples. (A) Number of read counts (upper), number of genes (middle), and percentage of mitochondrial gene counts (lower) detected in each sample. (B) Scatter plots show the correlation between the number of read counts and the number of genes detected in each sample. (C) UMAP plots of cells in each sample. (D) Heatmap showing the scaled, average expression of typical marker genes for cell types annotated in Figure 1B.

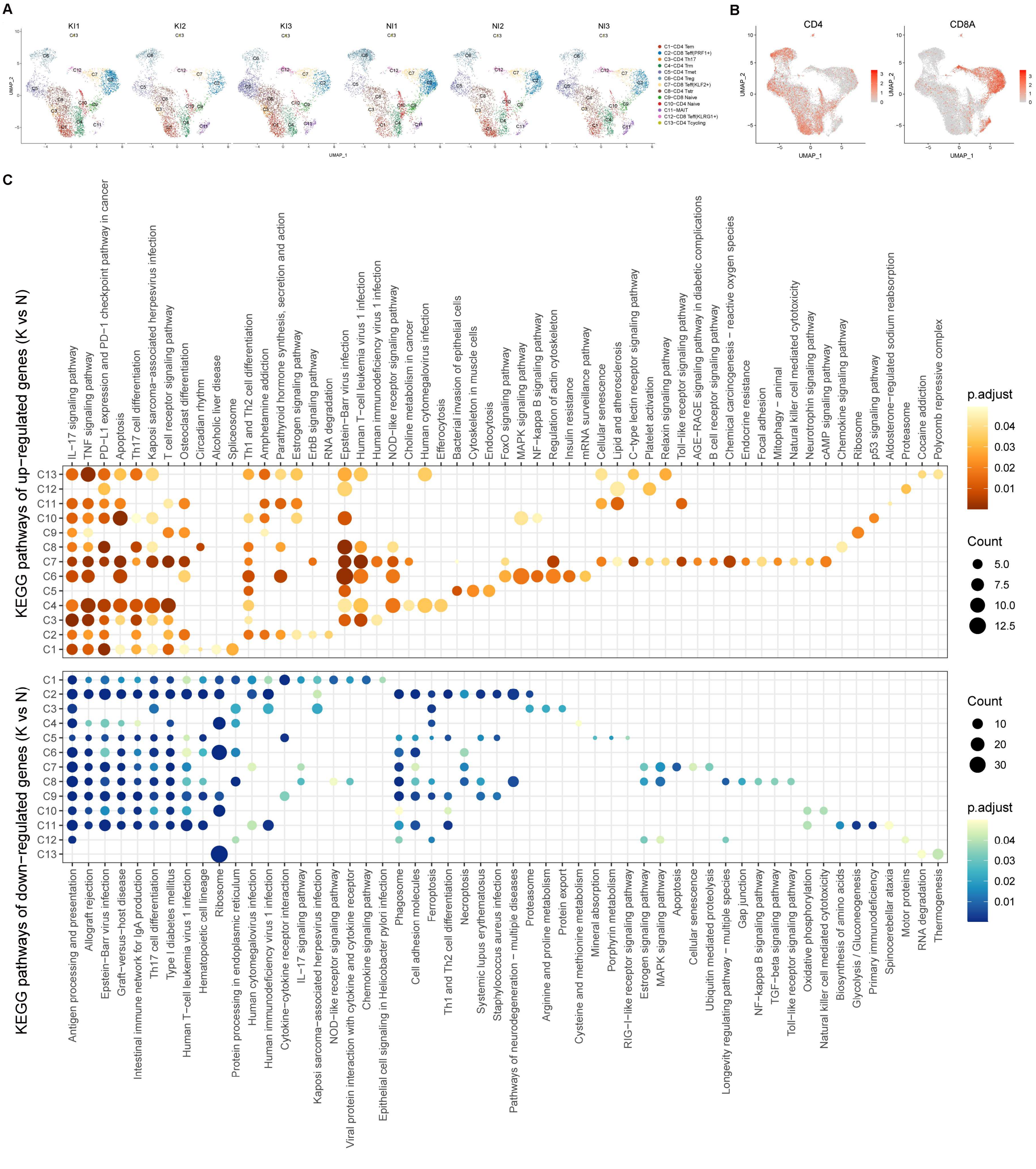

Supplementary Figure S2. Enrichment of subclusters in CD4$^+$ and CD8$^+$ T cells. (A) UMAP plots of T cell subclusters in each sample. (B) Expression of CD4 and CD8 gene. (C). Enriched KEGG pathways based on up-regulated genes (upper) and down-regulated genes (lower) compare keloid samples to normal scar samples of each subclusters in CD4$^+$ and CD8$^+$ T cells.

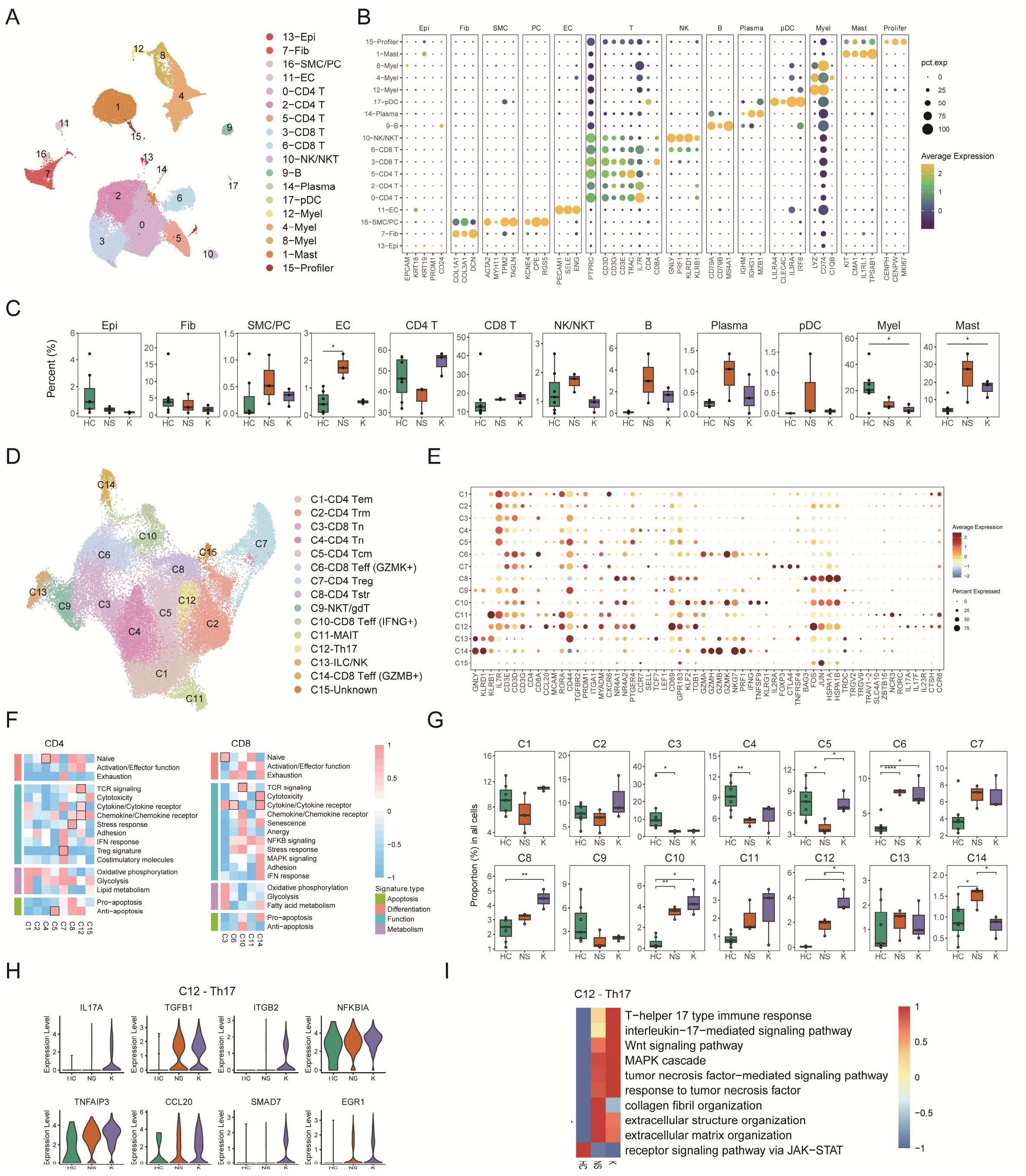

Supplementary Figure S3. Single-cell RNA-seq analysis of immune cells in keloid, normal scar and healthy skin samples. (A) Unsupervised clustering of the 73597 single cells from 3 keloid samples (17402 cells), 3 normal scar samples (23682 cells) and 7 healthy control skin (32513 cells), including 18 clusters. Epi: Epithelium, SMC/PC: Smooth Muscle Cell/Pericyte, Fib: Fibroblast, EC: Endothelial Cell, NK: Natural Killer, pDC: Plasmacytoid Dendritic Cell, Myel: Myeloid. (B) Dot plot of expression of key cell type marker genes in each cell cluster. Bubble size is proportional to the percentage of cells expressing a gene in a cluster and color intensity is related to

average scaled gene expression. (C) The percentage of cells in keloid, normal scar, and healthy control skin. Statistical analysis was performed using unpaired two-tailed t-tests. *: $p <=0.05$; K: Keloid, NS: Normal Scar, HC: Healthy Control. (D) UMAP (Uniform Manifold Approximation and Projection) of 15 subclusters identified in NK and T cells (46869 cells). Tem: effector memory T cell, Trm: resident memory T cell, Tn: naïve T cell, Tcm: central memory T cell, Teff: effector T cell, Treg: regulatory T cell, Tstr: T cell with stress response state, gdT: gamma-delta T cell, MAIT: mucosal-associated invariant T cell, ILC: Innate Lymphoid Cell. (E) Dot plots showing distinct expressions of the selected marker genes in each subcluster. (F) Heatmap illustrating the scaled score calculated based on expression of curated gene signatures across CD4+ T cell subclusters (left) and CD8+ T cell subclusters (right). (G) The box plots showing the percentage of cells for each subclusters of NK and T cells in keloid, normal scar, and healthy control skin samples. The p value indicated in the plot was calculated by unpaired two-tailed t-tests. (H) Violin plots showing differentially expressed genes of Th17 cells among keloid, normal scar, and healthy skin. (I) The pathways associated with fibrosis were scored in Th17 cells by AddModuleScore function among keloid, normal scar, and healthy control skin.

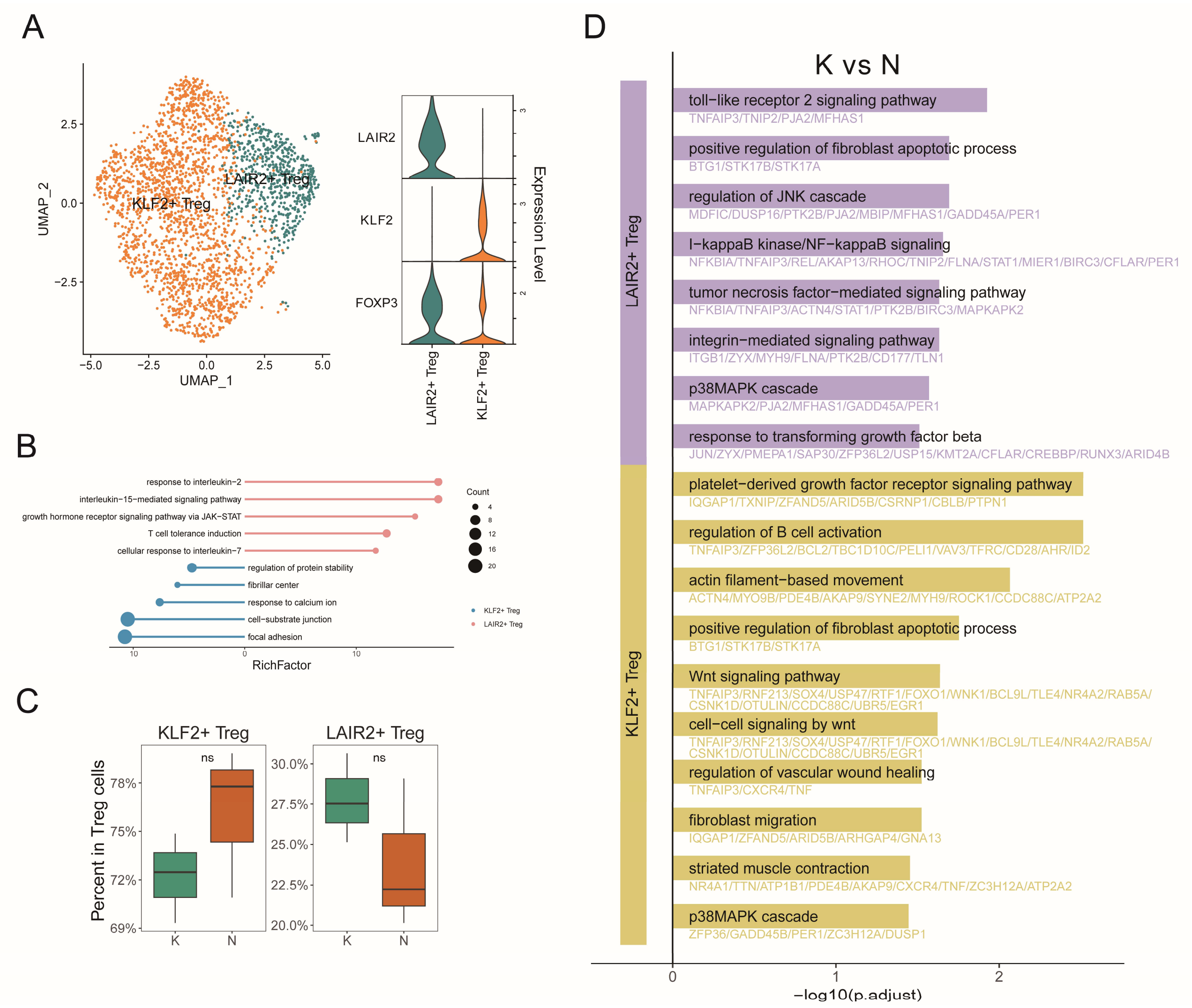

Supplementary Figure S4. Fibrotic skin disease and normal scar Tregs subcluster into distinct cell populations. (A) Subpopulation identified in Treg cells (left) and violin plot showing the expression of specific markers in Treg cell subpopulations (right). (B) Relative functional characteristics of Treg subpopulation. Pathways were enriched based on up-regulated genes between subpopulation comparison. (C) Box plot shows percentage of each subpopulation. (D) Pathway enriched with up-regulated gene (K vs N) in Treg subpopulations.

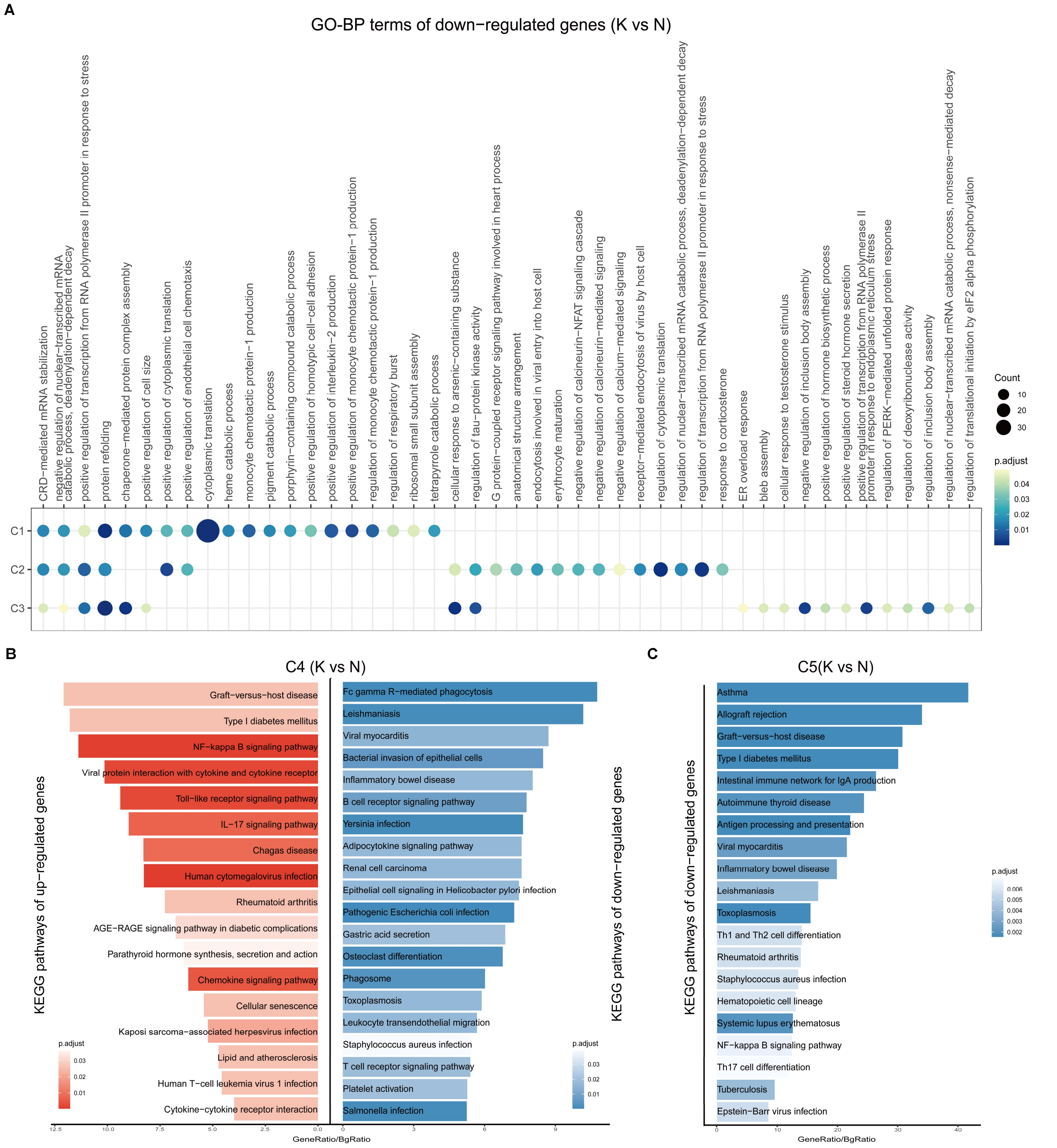

Supplementary Figure S5. Enrichment of subpopulation in mono-macrophages. (A) Enriched GO terms based on down-regulated genes (K vs N) of each subclusters in macrophage. (B) Enriched KEGG pathways based on up-regulated genes (left) and down-regulated genes (right) (K vs N) of CD14$^-$ Monocytes. (C) Enriched KEGG pathways based on down-regulated genes (K vs N) of CD14$^+$ Monocytes.

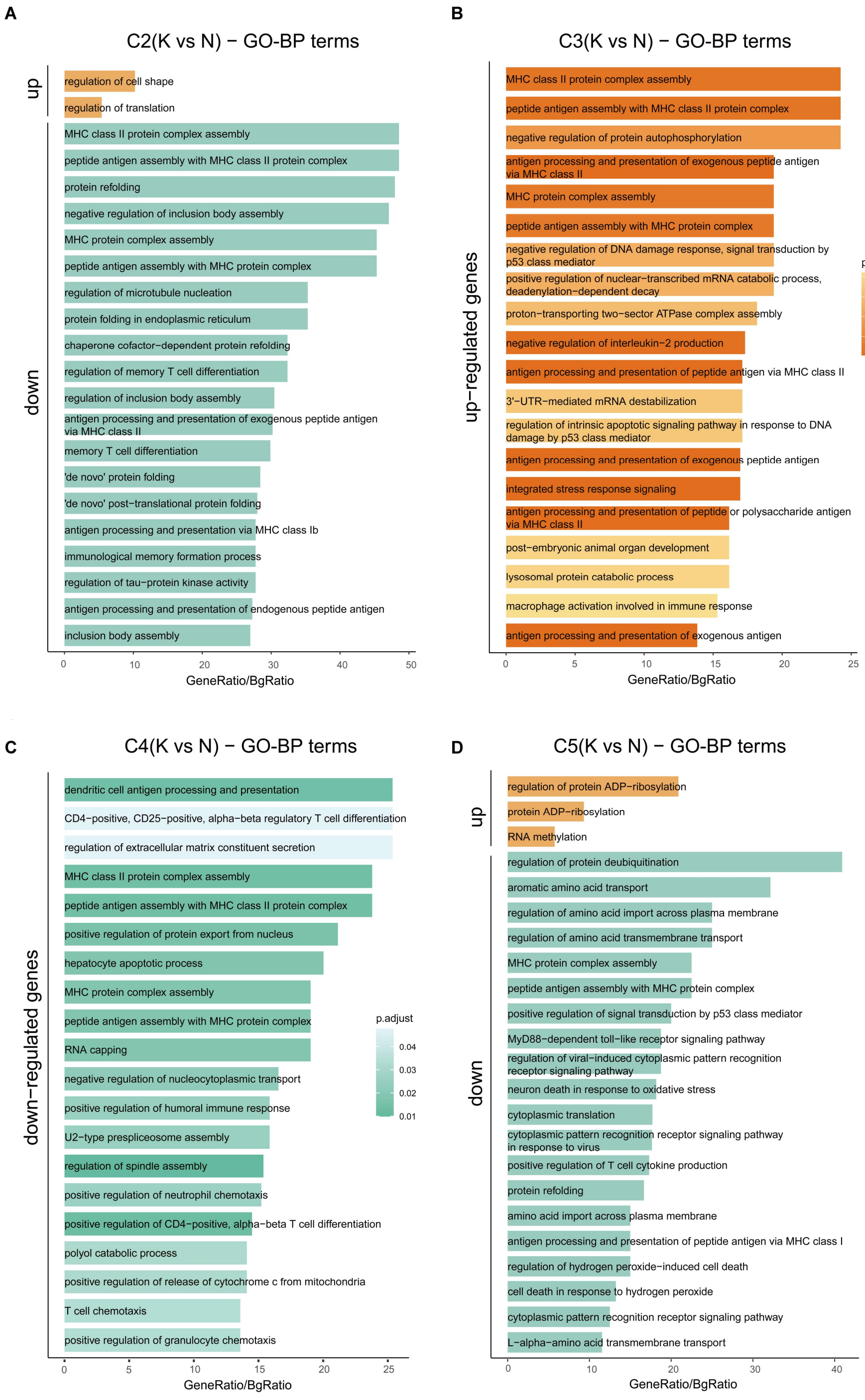

Supplementary Figure S6. Enrichment of each subcluster in DCs. (A) GO Biological Process enrichment analysis of differentially expressed genes between keloid and normal scar in C2 cluster. (B) GO analysis of up expressed genes compare keloid group to normal scar group in C3 cluster. (C) Enriched GO terms of down expressed genes compare keloid group to normal scar group in C4 cluster. (D) GO analysis of differently expressed genes in C5 cluster.

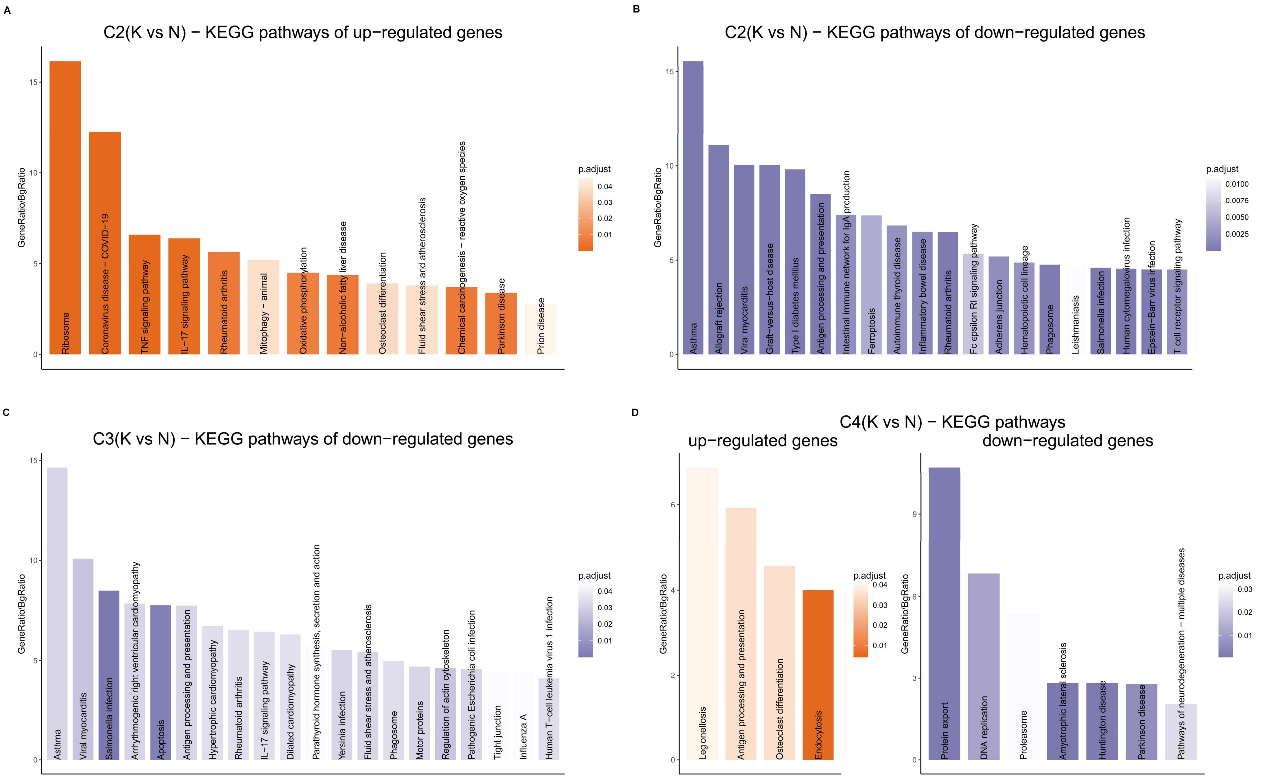

Supplementary Figure S7. Enrichment of each subcluster in mast cells. (A-B). KEGG pathways enriched with (A) up regulated genes and (B) down regulated genes in C2 subcluster. (C) Functional enrichment based down expressed genes in C3 subcluster. (D) KEGG analysis of differently expressed genes in C4 subcluster.

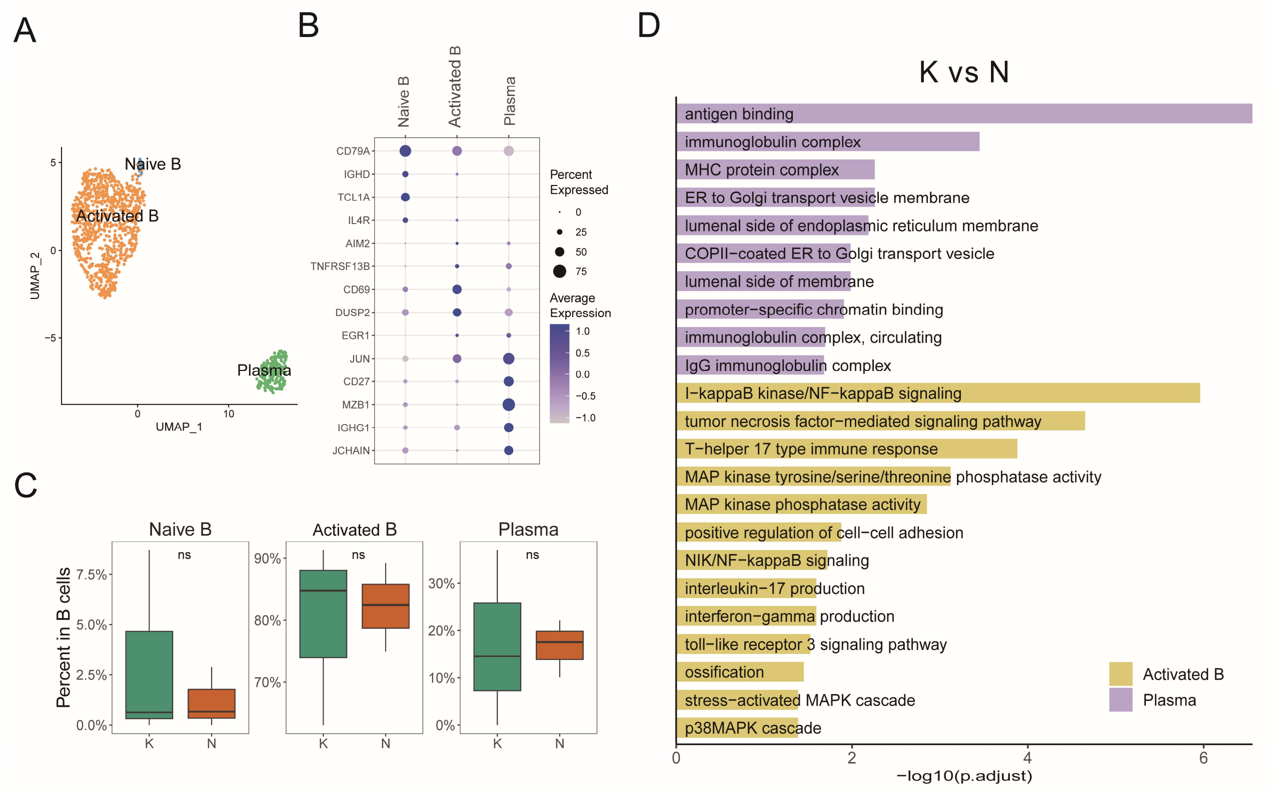

Supplementary Figure S8. Fibrotic skin disease and normal scar B cells subcluster into distinct cell populations. (A) Subpopulation identified in B cells. (B) Dot plot showing the expression of specific markers in B cell subpopulations. (C) Percentage change of each subpopulation. (D) Pathway enriched on up-regulated gene (K vs N) in Actiavted B cell and Plasma.

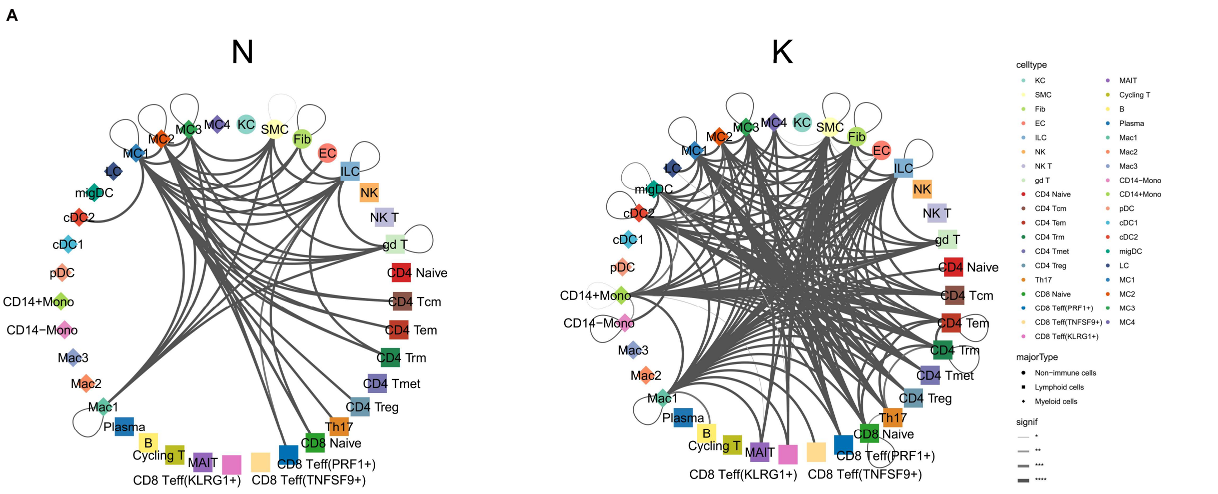

Supplementary Figure S9. Cellular interactions among all cell types in normal scar and keloid. (A) CSOmap analysis showing the interaction among all cell types of normal scar (left) and keloid (right). Line thickness represents the significance of cell-cell interaction.

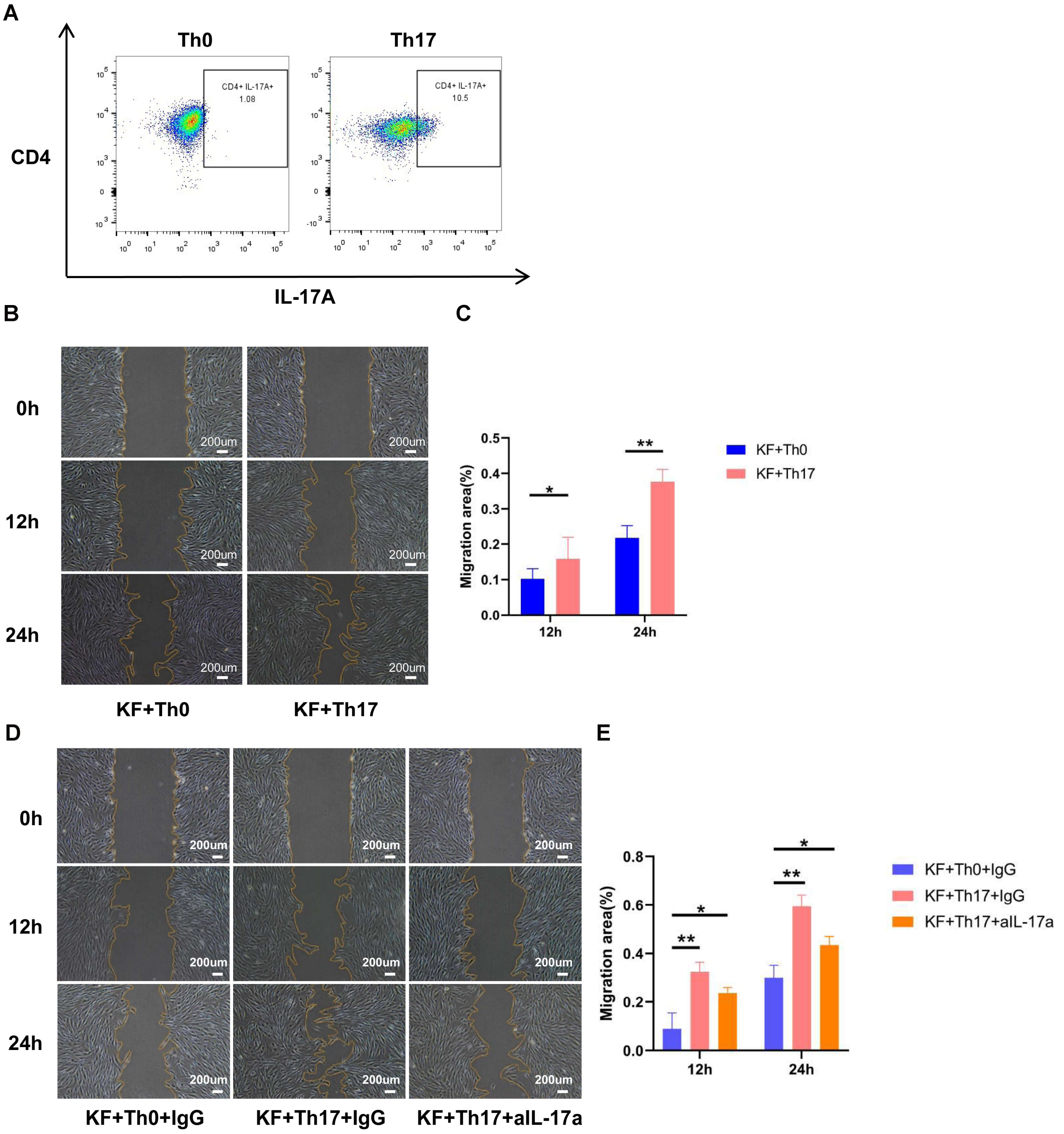

Supplementary Figure S10. Th17 cell promotes migration of keloid fibroblast through secreting IL-17A.（A）Flow cytometry representation depicting cultured Th17 cells in vitro.（B-C）Cell migration analysis of fibroblast co-cultured with Th0 or Th17 cells. Scale bar = 200μm. Error bars represent SD (n=3). *P<0.05 and **P<0.01.（D-E）Cell migration analysis of fibroblast co-cultured with Th0 or Th17 cells in the presence or absence of anti-IL-17A Ab. Error bars represent SD (n=3). *P<0.05 and **P<0.01.
